# In situ formation of mononuclear complexes by reaction-induced atomic dispersion of supported noble metal nanoparticles

**DOI:** 10.1038/s41467-019-12965-1

**Published:** 2019-11-21

**Authors:** Siquan Feng, Xiangen Song, Yang Liu, Xiangsong Lin, Li Yan, Siyue Liu, Wenrui Dong, Xueming Yang, Zheng Jiang, Yunjie Ding

**Affiliations:** 1 Dalian National Laboratory for Clean Energy, Dalian Institute of Chemical Physics, Chinese Academy of Sciences, 116023 Dalian, China; 2 University of Chinese Academy of Sciences, 100049 Beijing, China; 3 Shanghai Synchrotron Radiation Facility, Shanghai Institute of Applied Physics, and shanghai Advanced Research Institute, Chinese Academy of Sciences, 201204 Shanghai, China; 4 School of Materials and Textile Engineering, Jiaxing University, 314001 Jiaxing, China; 5 State Key Laboratory of Molecular Reaction Dynamics, Dalian Institute of Chemical Physics, Chinese Academy of Sciences, 116023 Dalian, China; 6 Hangzhou Institute of Advanced Studies, Zhejiang Normal University, 311231 Hangzhou, China; 7 State Key Laboratory of catalysis, Dalian Institute of Chemical Physics, Chinese Academy of Sciences, 116023 Dalian, China

**Keywords:** Catalyst synthesis, Heterogeneous catalysis, Synthesis and processing

## Abstract

Supported noble metal nanoclusters and single-metal-site catalysts are inclined to aggregate into particles, driven by the high surface-to-volume ratio. Herein, we report a general method to atomically disperse noble metal nanoparticles. The activated carbon supported nanoparticles of Ru, Rh, Pd, Ag, Ir and Pt metals with loading up to 5 wt. % are completely dispersed by reacting with CH_3_I and CO mixture. The dispersive process of the Rh nanoparticle is investigated in depth as an example. The in-situ detected I• radicals and CO molecules are identified to promote the breakage of Rh-Rh bonds and the formation of mononuclear complexes. The isolated Rh mononuclear complexes are immobilized by the oxygen-containing functional groups based on the effective atomic number rule. The method also provides a general strategy for the development of single-metal-site catalysts for other applications.

## Abstract (web summary)

Supported noble metal nanoclusters and single-metal-sites catalysts are inclined to aggregate into particles. Here, the authors report a general method with CO and CH_3_I to disperse the metal nanoparticles of Ru, Rh, Pd, Ag, Ir and Pt completely into single atoms with loading up to 5 wt%.

## Introduction

Supported noble metal catalysts have broadly been used in various heterogeneous catalytic reactions. However, deactivation is often an inevitable occurrence^1^. This can be caused by poisoning, coking, and sintering of metal nanoparticles (NPs). The agglomeration of NPs is one of the most important reasons^2–4^. The high ratio of coordinately unsaturated sites and boundaries exposed in small NPs would increase the surface energy and make it more active. Therefore, the driving force for sintering is the thermodynamic instability of small crystallite particles with a high surface-to-volume ratio, and it increases exponentially with the decrease of particle size^5,6^. The strong interaction between the reactants and metal NPs could overcome the interatomic metal–metal bond and facilitate the detachment of mononuclear metal complex from NPs. But the detached unstable monomer would agglomerate and reconstruct into larger particles again to lower their surface energy if there are no ligands to coordinate with the monomer or anchoring site to immobilize the monomer^7,8^. Thus large particles often grow up at the expense of small one by Ostwald ripening process.

Consequently, many approaches to reverse the sintering process by dispersing noble metal NPs have been developed^9^. Some common techniques are oxidation and reduction^10,11^, chlorination, and oxychlorination^12,13^, thermal treatment with halohydrocarbons^14–18^. Dispersion of large particles through the oxidation and reduction or oxychlorination often need high temperature in the range of 773–1073 K^9–13^. Therefore, these methods require the robustness and chemical inertness of the support. The operating conditions of the thermal treatment with halohydrocarbons are relatively mild. Hardacre et al. demonstrates that large NPs of Au supported on activated carbon (Au/AC) can be dispersed to single atom, dimer and trimer by CH_3_I during the induction period of vapor methanol carbonylation reaction under 513 K and 16 bar^14^ or via bromohydrocarbons or iodohydrocarbons treatment at atmospheric pressure in the temperature range of 323–513 K^15^. In addition, Hardacre et al. also investigate the method of using CH_3_I to disperse Au NPs supported on oxide support and discover that there is a drop in average NPs size after the treatment^16^. Thermal treatment with methyl iodide has been proven to be a facile and successful way to redisperse NPs^9^. However, the dispersion technique is mainly focused on Au and the improved method may be expanded to other noble metals with high loading.

Although the great progress has been made, the dispersion methods of NPs still face several challenges at present: (i) the operation conditions are usually harsh for many developed methods; (ii) there lacks a universal and simple approach to disperse most noble metal; (iii) most importantly, the degree of dispersion is not high enough for the subsequent process due to the limitation of Ostwald ripening. The atomic dispersion techniques of NPs not only achieves the maximum dispersion of NPs, but also serves as an effective way to fabricate heterogeneous single-metal-site catalysts^19–21^. Heterogeneous mononuclear complex catalyst can serve as a substitute for NPs and homogeneous catalysts, providing an ideal model to understand the catalytic mechanism at the molecular level^22^. A lot of advance works about supported mononuclear complex have been made by Gates et al. and many catalytic reactions are broadly explored by supported mononuclear complex catalyst^23–28^.

Here, we report a general atomic dispersion technique of supported noble metal nanoparticles with loading up to 5 wt%. The nanoparticles of most precious metals supported on activated carbon (AC), such as Ru, Rh, Pd, Ag, Ir, and Pt are completely atomically dispersed in the form of mononuclear complex by a reaction with a mixture of CH_3_I and CO. The dispersive process is detailedly discussed in the case of Rh metal as an example.

## Results

### Discovery of Rh NPs converting to mononuclear complexes

The AC supported 5 wt.% Rh NPs was first prepared by the calcination of AC supported rhodium chloride in a flow of N_2_, and then the subsequent reduction by H_2_ (denoted as Rh/AC). Then the Rh/AC was heated up to 513 K in a flow of N_2_, then switched into a flow mixture of CO and CH_3_I (denoted as CO/CH_3_I) and then maintained for 6 h. Then the obtained catalyst was cooled to room temperature in a flow of N_2_ or CO for various characterizations (denoted as Rh_1_/AC). All the experiments were conducted at atmospheric pressure (see Methods).

**Fig. 1:**
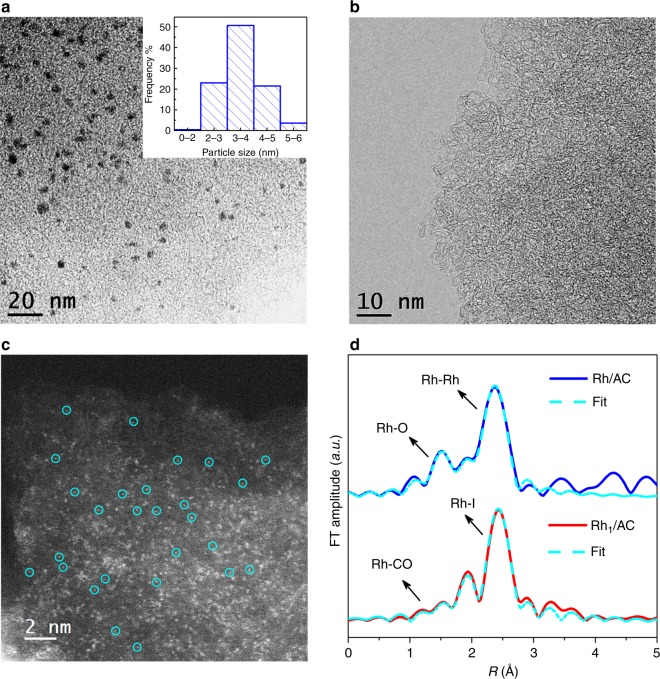
Structure of the Rh/AC and Rh_1_/AC catalysts. **a** The HRTEM image of Rh/AC and the corresponding particle size distribution. **b** The HRTEM and **c** HAADF-STEM image of Rh_1_/AC. **d** Comparison of the experimental (solid line) and fitted (dashed line) curves of *k*^2^-weight EXAFS spectra of Rh/AC and Rh_1_/AC catalysts

TEM image and the corresponding particle size distribution (Fig. 1a) show that the size of Rh NPs centered at 4 nm in the Rh/AC catalyst. In the XRD patterns of Rh/AC (Supplementary Fig. 1), the peaks at about 41°, 47°, 70°, and 85° are attributed to the diffraction of (111), (002), (022), and (113) lattice planes of cubic Rh particle. The calculated average crystal particle size from XDR patterns is ~5 nm, indicating the presence of a portion of big nanocrystal. After the treatment by CO/CH_3_I, neither appeared peaks belonged to Rh NPs in the XRD patterns (Supplementary Fig. 1), nor could Rh NPs be observed on TEM image (Fig. 1b) over the Rh_1_/AC. Furthermore, atomic energy dispersive spectroscopy (EDS) mapping (Supplementary Fig. 2) shows the uniform distribution of Rh atom on Rh_1_/AC. Additionally, isolated Rh ions or atoms were predominated on the Rh_1_/AC according to the high-angle annular dark-field scanning transmission electron microscopy (HAADF-STEM) images (circled in Fig. 1c). Due to the small difference in atomic number between I (*Z* = 53) and Rh (*Z* = 45), the iodine atoms might disturb the recognition of the single Rh atoms in HAADF-STEM. In order to further confirm the sole existence of Rh single atoms on Rh_1_/AC, extended X-ray absorption fine structure (EXAFS) was performed to estimate the average coordination number. EXAFS peaks originally belonged to Rh–Rh (2.69 Å) on Rh/AC with a coordination number of 5.0 disappeared, while the fitted coordination numbers of Rh–Rh shells were close to zero on Rh_1_/AC. As is shown in the fitting result (Fig. 1d, and Supplementary Fig. 3 and Supplementary Table 1), the bond length of Rh–Rh is very close to that of Rh–I, which makes it difficult to distinguish the two bonds in *R* space. However, wavelet transform (WT) analysis can provide more accurate and full-scale information for separating backscattering atoms in both radial distance and *k*-space resolution^29,30^. From the WT contour plots of Rh/AC and Rh_1_/AC in Supplementary Fig. 4, it can be clearly seen that although the *R* values of the maximum intensity are quite similar, the *k* values of 8.1 Å^−1^ for Rh/AC and 10.9 Å^−1^ for Rh_1_/AC can be assigned to Rh–Rh and Rh–I coordination shell, respectively. No signal of Rh–Rh backscattering in *k* space can be observed in Rh_1_/AC, which further confirms the complete transformation of Rh NPs into Rh single-atom sites. Three peaks at 2.67, 2.09, and 1.88 Å were fitted to Rh–I, Rh–O–AC (oxygen-containing functional group on the surface of the AC) and Rh–CO distance with a coordination number of 3.7, 1.0, and 1.7, respectively (Fig. 1d, Supplementary Fig. 3 and Supplementary Table 1). The observed coordination number of Rh–CO in Rh_1_/AC was roughly consistent with the CO/Rh molar ratio of 1.36 which was obtained by CO temperature-programmed desorption (Supplementary Fig. 5). The attenuated total reflectance-Fourier transform infrared (ATR-FTIR) spectroscopy (Supplementary Fig. 6) shows the peaks attributed to [Rh(CO)I_4_] at ca. 2160 cm^−1^ and [Rh(CO)_2_I_3_] at ca. 2030 and 2017 cm^−1^, respectively^31^. X-ray photoelectron spectroscopy (XPS) analysis (Supplementary Fig. 7 and Supplementary Table 2) shows dominant Rh^3+^ species existed in Rh_1_/AC. CO (m/z = 28), Rh (m/z = 102.9) and I^•^ (m/z = 127) species were detected by laser desorption ionization/time of flight mass spectrometer (LDI/TOF-MS) on Rh_1_/AC (Supplementary Fig. 8). Based on these observations, we can conclude that the atomic dispersion Rh exists as the form of dominating Rh(CO)_2_I_3_(O–AC) and a portion of Rh(CO)I_4_(O–AC) in Rh_1_/AC. Using our approaches, little metal loss was detected in the process, confirmed by inductively coupled plasma optical emission spectrometer (ICP-OES) analysis (Supplementary Table 3). In addition, this method is simple for large-scale operation and tens of kilograms of Rh/AC have been easily atomically dispersed in our laboratory.

### Atomically dispersive process and mechanism of Rh NPs

**Fig. 2:**
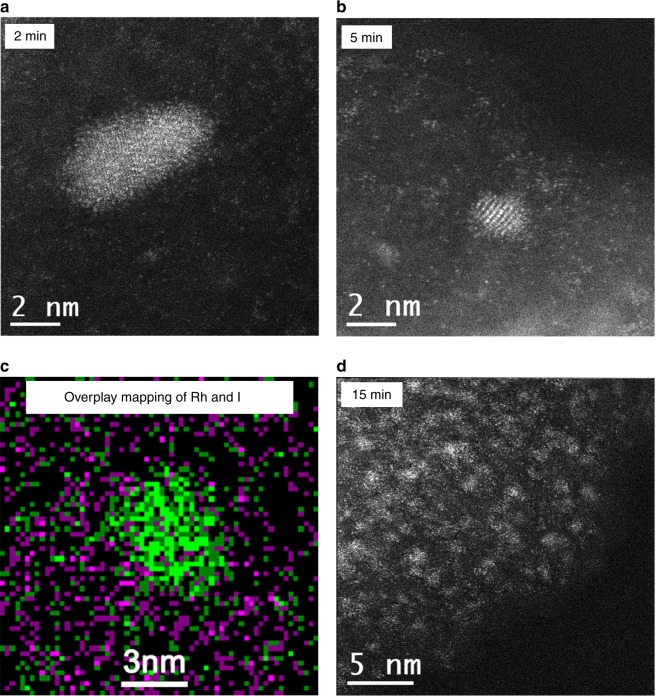
Time resolution HAADF-STEM pictures of Rh/AC dispersion. The sample treated by CO/CH_3_I at 513 K for **a** 2 min, **b** 5 min, and **c** the corresponding Rh and I atomic EDS mapping of Fig. 2**b**, **d**, 15 min. Green color denotes Rh element and pink color denotes I element

**Fig. 3:**
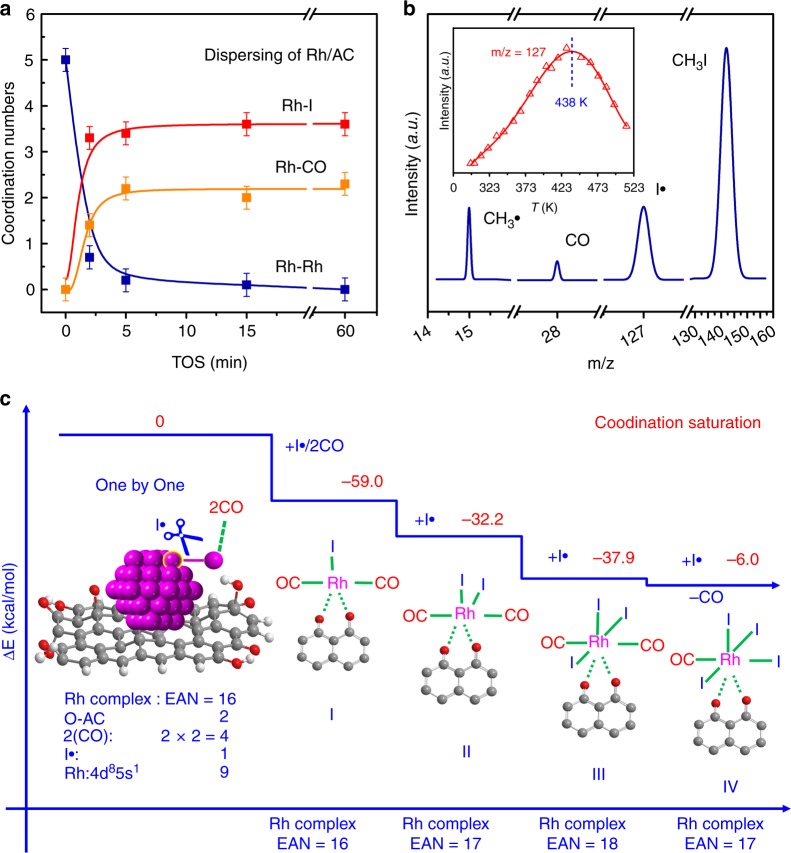
Structure and model for the atomically dispersive process of Rh NPs. **a** Coordination number variation of Rh–Rh, Rh–I, and Rh–CO during atomically dispersion process derived from EXAFS result (Supplementary Table 4) of Rh/AC treated with CO/CH_3_I at 513 K for different time. **b** The representative mass spectra of different reactant and radical species detected during the in-situ reaction between Rh/AC and CO/CH_3_I at 513 K by LDI/TOF-MS. The inset is the variation of iodine radicals (I^•^) signals during the reaction between Rh/AC and CO/CH_3_I at different temperature. **c** The atomic dispersion model of Rh NPs via one-by-one mechanism based on the 16–18 effective atomic number (EAN) rule

To investigate the Rh atomically dispersive process, we treated the Rh/AC sample with CO/CH_3_I mixture for different time durations. The HAADF-STEM image of 2 min time on stream (TOS) displayed NP ~4–5 nm (Fig. 2a). After 5 min, sub-NP with single atoms scattered around still existed (Fig. 2b), consistent with the corresponding atomic EDS mapping (Fig. 2c). Multimers, trimers and dimers account for the majority after 15 min treatment (Fig. 2d). These images show that the size of NPs decreased little by little. The quantitative analysis of EXAFS data shows that the coordination number of Rh–Rh decreased from 5.0 to 0.7, 0.3 and 0 after 2, 5, and 15 min treatment, individually (Fig. 3a, Supplementary Fig. 9 and Supplementary Table 4). On the contrary, the coordination number between Rh and I increased from 0 to 3.3, 3.3 and 3.6 with 2, 5, and 15 min TOS, respectively, and then kept at ~3.6. The variation of Rh–CO with TOS was similar to that of Rh–I. The similar trends could be observed in XRD experiment (Supplementary Fig. 10). The diffraction peaks associated with cubic-Rh NPs decreased sharply over a period of 2 min and only little features associated with cubic-Rh were found after 5 min. After reaction for 15 min, the diffraction peaks belonged to cubic-Rh completely disappeared. These results suggested the gradual shrinkage of the large Rh NPs due to the continued removal of the exfoliated monomer by CO on the surface of AC support.

In theory, the cleavage reaction of CH_3_I could occur on the surface of the metal, yielding a large amount of free radicals^32^. The signals of methyl radical (CH_3_∙) and iodine radicals (I∙) can be detected if the quenching of short-lived radicals can be retarded at ultralow pressure or by the high dilution of the feed gas. The short-lived radicals were also observed in the in-situ LDI/TOF-MS experiment when CO/CH_3_I passed through the Rh/AC. As shown in Fig. 3b, CH_3_^•^ (m/z = 15) and I^•^ (m/z = 127) radicals accompanied with CO (m/z = 28) and CH_3_I (m/z = 142) were clearly distinguished in the spectra, implying the homolytic cleavage of CH_3_I on the surface of Rh NPs. While the signals of Rh carbonyl species could not be found, indicating that the migration of the Rh carbonyl species occurred on the surface of AC^7^. In general, high temperature favors the dissociation of CH_3_I. However, when the dispersion of NPs started at certain temperature in the form of mononuclear complexes, I• radicals would be continuously consumed and the number of metal NPs also decreased. Therefore, the signal intensity of I• radicals would show a maximum point with the increase of temperature. In fact, the signal variation with temperature (inset of Fig. 3b) shows that the intensity of I^•^ radicals reached maximum at 438 K and then decreased quickly from 438 K to 513 K, suggesting the crucial role of I^•^ radical in the cleavage of Rh–Rh bond and the formation of mononuclear complexes. Further experiment confirmed that the NPs on Rh/AC could also be atomically dispersed at ca. 438 K when both CO and CH_3_I existed (Supplementary Fig. 11a), but failed at 373 K (Supplementary Fig. 11b).

In addition, the similar particle size distribution remained on Rh/AC catalyst after individual treatment by CO (Supplementary Fig. 1 and Supplementary Fig. 12a) or CH_3_I/N_2_ (Supplementary Fig. 1 and Supplementary Fig. 12b) at 513 K for 6 h, indicating that the sole action of CO or CH_3_I could not atomically disperse Rh NP. During the process of Ostwald ripening, the detached monomer would agglomerate into larger particles if there were no anchored site to immobilize the monomer. In order to explore the effect of O–AC for the atomic dispersion, the AC samples were treated under H_2_ atmosphere at 1273 K to remove O–AC. After treated with H_2_, the NMR peaks at 216 and 181 ppm belonged to carbonyl and the peak at 121 ppm ascribed to benzene ring all disappeared, indicating the elimination of almost all oxygen-containing functional groups (Supplementary Fig. 13). The TPD-MS further indicates that no O–AC decomposed during the temperature-rise period on the H_2_ treated AC (Supplementary Fig. 14). Without these oxygen-containing functional groups, the Rh NPs became even bigger after treated by CO/CH_3_I, which followed Ostwald ripening^33,34^ (Supplementary Fig. 15). This indicated the crucial role of O–AC for the atomic dispersion of Rh NPs. Accordingly, the combined actions of proper temperature, CO, CH_3_I, and O–AC are indispensable for the atomic dispersion of Rh NPs.

Various oxygen-containing functional groups can be found on the surface of AC, such as carboxyl, lactone, anhydride, phenol, carbonyl, and ether^35^. Carboxyl, lactone, and anhydride are unstable and decompose at low temperature in the form of CO_2_ (Supplementary Fig. 14). While the phenol, carbonyl, and ether are relatively stable. Interestingly, a carbon vacancy along with either isolated phenol group or adjacent phenol-carbonyl pairs in the reduced graphene oxide surface served as anchored site for single Pd atom by atomic layer deposition^36,37^. In the AC supported single sites catalyst for heterogeneous carbonylation, a broken ether bond was proposed as the site to immobilize the single metal ion^38^. For the modeling of DFT calculation, we chose carbonyl and ether as the anchored site for mononuclear complex (Supplementary Fig. 16) based on the key role of carbonyl and benzene groups on the surface of AC on the formation of mononuclear complexes.

**Fig. 4:**
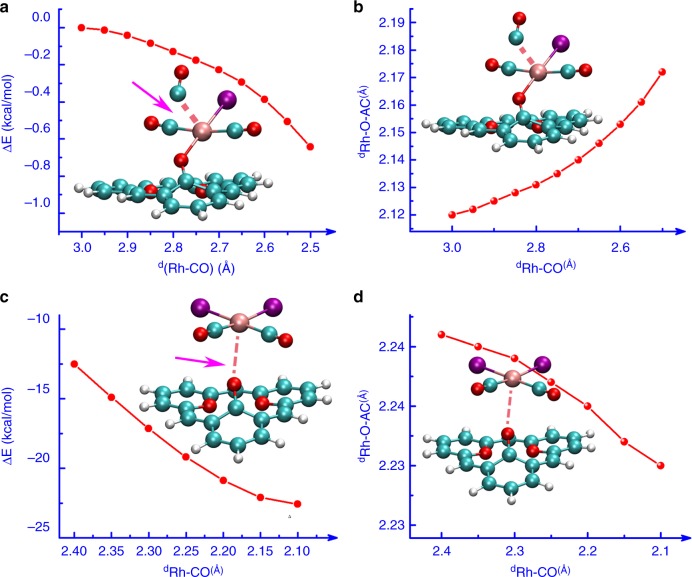
Dynamic simulation of Rh(CO)_2_I(O–AC) migration and transformation. **a**, **b** Variation of energy and Rh–O–AC bond distance during the process of Rh(CO)_2_I(O–AC) attacked by CO. **c**, **d** Variation of energy and Rh–O–AC bond distance during the process of Rh(CO)_2_I_2_ anchoring on the neighboring oxygen site in the surface of AC

Density function theory (DFT) calculations showed that the atomic dispersion of Rh NPs was accomplished via one-by-one mechanism based on the effective atomic number (EAN) rule^39^ (Fig. 3c and Supplementary Fig. 17a). Once one atom of Rh NPs was attacked by CO and I• free radical (Fig. 3c and Supplementary Table 5), the Rh atom was extracted as Rh(CO)_2_I(O–AC) complex from the metal NPs with a lower system energy of −59.0 kcal/mol. Furthermore, the dispersion process of Rh NPs to take the interactions between Rh and CO/I• free radical is spontaneous without transition state (Supplementary Fig. 17b). However, the structure of Rh(CO)_2_I(O–AC) complex was not the most stable one. The dynamic simulation of Rh(CO)_2_I (O–AC) attacked by CO shows that the formation of Rh(CO)_3_I would bring about the breakage of the bond between Rh and O–AC (Fig. 4a, b). On the contrary, after attacked by I• free radical, the anchoring of Rh(CO)_2_I_2_ on the neighboring oxygen site is favorable, based on the change of both energy and bond distance (Fig. 4c, d). These results manifested that the formation of Rh(CO)_2_I_2_(O–AC) was easier than that of Rh(CO)_3_I(O–AC). Furthermore, the I• free radical could further attack the virtual π* orbital of Rh atom to form more stable Rh(CO)_2_I_3_(O–AC) structure or even substitute a CO molecule to form Rh(CO)I_4_(O–AC) via the Rh(CO)_2_I_2_(O–AC) intermediate with the reactions enthalpies of −70.1 and −76.1 kcal/mol, respectively (Fig. 3c and Supplementary Fig. 18). Accordingly, the atomic dispersion of AC supported Rh NPs is favorable in thermodynamics when both CO and CH_3_I participate concurrently in the reaction due to the strong exothermicity.

### Universality of atomic dispersion for noble metal NPs

**Fig. 5:**
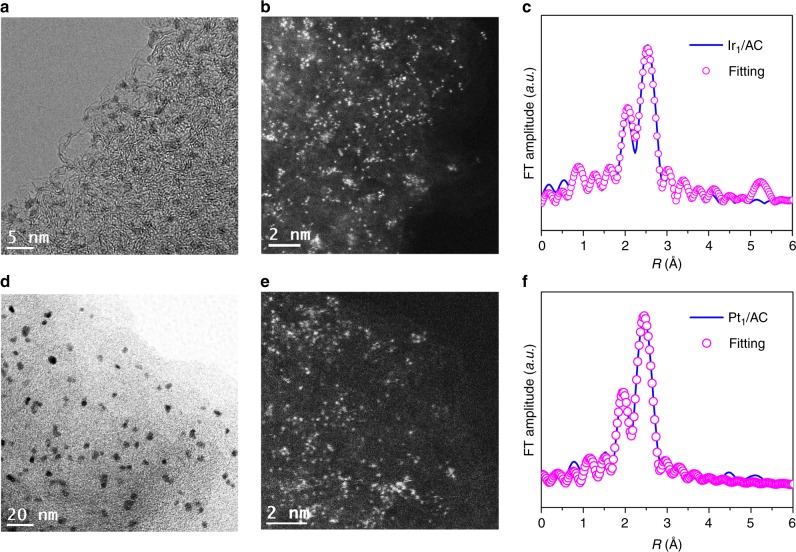
Atomic dispersion of supported Ir and Pt NPs. **a** The HRTEM image of Ir/AC; **b** the HAADF-STEM image of Ir_1_/AC; **c** comparison of the experimental (solid line) and fitted (dashed line) curve of *k*^2^-weight EXAFS spectra of Ir_1_/AC catalyst. **d** The HRTEM image of Pt/AC; **e** the HAADF-STEM image of Pt_1_/AC; **f** comparison of the experimental (solid line) and fitted (dashed line) curve of *k*^2^-weight EXAFS spectra of Pt_1_/AC catalyst

Inspired by the phenomenon, we wonder whether the strategy of atomic dispersion was also applicable for other noble metals. Surprisingly, the NPs of Ru, Pd, Ag, Ir, and Pt all could be dispersed to the corresponding single-metal-sites after the treatment of CO/CH_3_I. The TEM and HAADF-STEM pictures in Fig. 5a, b, d, e show that the NPs of Ir and Pt were also successfully dispersed to single atom. The EXAFS spectra after dispersion indicated that the Ir–Ir bonds in Ir NPs and the Pt–Pt bonds of Pt NPs both disappear (Fig. 5c, f and Supplementary Table 6). The single-metal-site of Ir_1_/AC and Pt_1_/AC also exist in the form of mononuclear complex, similar to that of Rh_1_/AC. Similar phenomenon was observed on the Ru/AC (Supplementary Fig. 19), Pd/AC (Supplementary Fig. 20) and Ag/AC (Supplementary Fig. 21) samples after the CO/CH_3_I similar thermal treatment. Almost all the noble metal NPs could have underwent reverse agglomeration processes and dispersed into the isolated mononuclear complexes in the presence of CH_3_I/CO mixture. These results demonstrated that the atomic dispersion of supported noble metal nanoparticles was not an individual phenomenon but a shared route to disintegrate metal NPs to single-metal-sites.

## Discussion

A simple and quick protocol for the atomic dispersion of precious metal NPs has been developed. In the case of Rh metal, the combined action of a certain temperature and presence of CO, CH_3_I, and O–AC were indispensable for the atomic dispersion of Rh NPs. This universal approach could be applicable for most noble metals and also easy for large-scale production. The desired dispersion of NPs can also be further obtained by controlling the post processing conditions of the supported mononuclear complexes^25^, achieving the goal of catalyst regeneration. Furthermore, supported mononuclear complexes with the maximized atom efficiency up to 100% exhibited unique catalytic performance^22^. The simplicity and uniformity of supported mononuclear complexes offer the advantage of structure determination of active sites dispensable of ultrahigh vacuum. The composition identification of surface metal complex, including ligands and metal-support bond facilitates the understanding of electron-donor effect for the catalyst reactivity and selectivity, leading to the deep comprehension of structure-performance correlations. Different from many reported fabrication method for the supported mononuclear complexes in which the catalytic active sites were prepared via the reactions of organometallic precursors with the support surface, this work provides a universal and simple strategy to synthesize supported mononuclear complexes of most precious metal. It will bring about new insights and points for the catalyst design.

## Methods

### Preparation of catalyst

The coarse coconut shell activated carbon (AC) of 40–60 mesh was washed with deionized water (353 K) till the electrical conductibility of washings was below 20 μs/cm, and afterwards dried at 393 K for 12 h (AC-washed). Some of which were calcinated in tubular furnace in a flow of H_2_ at 1273 K for 6 h to eliminate the oxygen-containing group on the surface of AC (AC-treated-H_2_). Taking Rh metal as an example, 1.35 g RhCl_3_∙*n*H_2_O with 37 wt.% metal content was dissolved into 15 mL deionized water, then 10 g AC-washed or AC-treated-H_2_ was impregnated, and then stirred continuously till no more bubbles could be discerned, and then dried in 363 K water bath. The sample was further dried at 393 K overnight and calcined at 573 K for 2 h in a flow of N_2_ (100 mL/min) in quartz tubular reactor. Then the calcined sample was treated with H_2_ at 573 K for 2 h to obtain the fresh 5 wt.% Rh catalyst, which was denoted as Rh/AC. The Rh/AC sample was heated from room temperature to 513 K in a flow of N_2_, and held for 20 min, then switched to CO/CH_3_I mixture (CO passed through a bottle filled with CH_3_I at 298 K, 30 mL/min) for 6 h, and cooled to room temperature in a flow of CO or N_2_. And the obtained catalyst was denoted as Rh_1_/AC. Other samples of Ru/AC, Pd /AC, Ag/AC, Ir/AC, and Pt/AC with 5 wt% metal loading were prepared and dispersed to Ru_1_/AC, Pd_1_/AC, Ag_1_/AC, Ir_1_/AC, and Pt_1_/AC as the similar procedures of that of Rh/AC and Rh_1_/AC, except that the Ir/AC sample was obtained by the reduction of syngas at 513 K for 2 h (CO/H_2_ = 4 volume ratio).

### Catalyst characterization

Inductively coupled plasma optical emission spectrometer (ICP-OES) measurement was performed with PerkinElmer ICP-OES 7300DV. The mixture solution of H_2_O_2_ and concentrated HNO_3_ was employed to dissolve the samples at high temperature and pressure in a sealed test bottle, and then aquaregia (HCl/HNO_3_ = 3/1vol) was added in to dissolve the residues.

X-ray diffraction (XRD) measurement was performed using PANalytical X’Pert Pro X-ray diffractometer with Cu Kα X-ray source at a wavelength of 1.5045 Å. X-ray photoelectron spectroscopy (XPS) characterization was performed using a Thermo fisher ESCALAB 250Xi, which is equipped with an Al Kα radiation X-ray source.

The high-resolution transmission electron microscope (HR-TEM) images of the samples were obtained with JEM-2100 equipped with energy dispersive spectrometer (EDS). Particle size distribution was evaluated based on the statistic result with the soft aid of Nano Measure from HRTEM images. The aberration-corrected high-angle annular dark-field scanning transmission electron microscopy (HAADF-STEM) images were also gained using a JEM-ARM200F STEM/TEM instrument with a CEOS probe corrector working at 200 kV to guarantee a resolution of 0.08 nm.

The obtained Rh HSMSC cooled in a flow of CO was characterized by temperature-programmed desorption-mass spectrum (TPD-MS) using AMI 300 instrument. The 200 mg sample was first pretreated at 323 K for 1 h in a flow of helium (30 mL/min) to remove species of physical adsorption. After the stabilization of baseline, TPD data were collected using a thermal conductivity detector (TCD) and mass spectrometer at a heating rate of 10 K/min to 1273 K. For quantification of total desorbed CO, the peak area of TCD signal was calibrated with pulsed sampling using standard gas of 10% CO/He.

Attenuated total reflectance Fourier transform infrared (ATR-FRIR) experiments were performed by depositing the fine powder of catalyst on top of a Ge internal reflection element (IRE). The spectra were recorded between 800 and 4000 cm^−1^ by averaging 128 scans at a resolution of 4 cm^−1^.

Extended X-ray absorption fine structure (EXAFS) data of Rh K-edge were collected at beamline BL14W1 of Shanghai Synchrotron Radiation Facility (SSRF). The data were recorded in the fluorescence mode equipped with Electro-Lyte detector. The original EXAFS data were analyzed by the Demeter software package. Fourier transformation was applied to process the k^3^-weighted raw data. The theoretical scattering amplitude and phase-shift functions of all the paths for fitting the EXAFS data were calculated with FEFF6 code. The Rh L_3_-edge X-ray adsorption near-edge structure (XANES) spectra were tested at the 4B7A beamline of the Beijing Synchrotron Radiation Facility (BSRF), China, in total electron yield (TEY) mode, where the sample drain current was collected under pressure smaller than 5 × 10^–8^ Pa.

Solid-state ^13^C magic angle spinning nuclear magnetic resonance spectroscopy (MAS NMR) experiment was operated on a VARIAN infinity plus spectrometer equipped with a 2.5-mm probe at a frequency of 161.8 MHz.

Laser desorption ionization/time of flight mass spectrometer (LDI/TOF-MS) experiments of Rh HSMSC were conducted to detect the laser dissociative fragment of Rh HSMSC and further identify its single complex structure, and the in-situ formed short-lived radical species were also detected with LDI/TOF-MS. The powder of Rh HSMSC sample was attached to an aluminum groove holder of 8 mm × 3 mm in the vacuum chamber. Laser light of 266 nm was slightly focused on the sample. The skimmed Ar atom beam passed up the sample surface with a distance of ~1 mm to carry the dissociated and sputtering substance to the detecting chamber. For the in-situ experiment, the Rh/AC sample was placed in the back-end of quartz tube reactor. The back-end was designed as an inverted conic shape which has a very small hole in the conic apex to diffuse reactant and radical species into detecting chamber. After introducing CO/CH_3_I into the reactor, the pressure of the reactor and chamber was kept at 10^−2^ and 10^−7^ Torr. CH_3_I steam was carried in with CO at ambient temperature. Then the Rh/AC sample was heated by electrical resistance trace heating with a heating rate of 4.0 K/min from room temperature to desired temperature in a flow of CO/CH_3_I mixture (CO passed through a bottle filled with CH_3_I at 298 K, 1 mL/min). The species in detecting chamber would be ionized by 118 nm radiation. The resultant ions were analyzed by time of flight mass spectrometry equipped with a microchannel plate detector.

### Density function theory calculation

The molecule structures were optimized by the density function theory (DFT) calculation using Gaussian 09 package under gas-phase condition^40^. The generalized gradient approximation (GGA) of the exchange and correlation function was calculated using B3LYP method^41^ with grime dispersion correction (GD3)^42^. We adapted a more accurate basis set, def2-TZVPD^43^ for Rh and I atoms and 6–31 + G** for C, H, and O atoms. The stable geometries of all Rh complexes were verified by frequency calculations with no imaginary frequencies. The molecule orbitals (MO) analysis was based on the same basis which produces a small variation of MO. The natural bond order analysis also was performed both with B3LYP function and with 6–311 + + G** for C, H, and O atoms. In this study, the unit of molecule bond length or distance between atoms is angstrom.

In order to reasonably show the role and function of AC in our study system, the carbonyl models of AC have been established and optimized using the B3LYP method. For purpose of studying the dispersion behavior of Rh atom from the surface of Rh NPs, the geometric structures and the energies were calculated with Vienna ab initio simulation package (VASP)^44–47^. The Perdew-Burke-Ernzerhof (PBE) was used as the exchange correlation function^48^. The cut off kinetic energy was set to 420 eV. Brillouin zone integration was approximated using the Monkhorst-Pack *k*-points method^49^ and set as 1 × 1 × 1. The structure models were created with the isolated systems and the side length of simulation box was selected with 36 Å to eliminate the size error. Geometries were optimized until the energy was converged to 1.0 × 10^–4^ eV/atom and the force was converged to 0.05 eV/Å. Here, it is need to note that the Rh1 atom was randomly selected on the surface of Rh NPs to clearly show the bond changes between Rh_1_ atom and neighbor nine Rh atoms. To understand and account for dispersion effect in the Rh NPs dispersion process, the energy corrections of van der Waals (vdW) were calculated using the semiempirical approach proposed by Grimme^50^ (DFT-D2 method) in conjunction with the PBE functional. All DFT-D2 geometry optimizations of Rh NPs complexes were performed starting from the PBE optimized structures without vdW correction.

## Supplementary information

Supplementary Information

Peer Review

Source Data

## Data Availability

The data that support the findings in this study are in the published article and/or its Supplementary Information files. The data sources are deposited on the generalist repository of figshare, and the accession code is https://doi.org/10.6084/m9.figshare.9948161. The whole datasets are available from the corresponding author on reasonable request. The source data underlying Figs. 1–5 and Supplementary Figs. 1–21, and Supplementary Tables 1–6 are provided as a Source Data file.
