# Supplementary material for: In situ formation of mononuclear complexes by reaction-induced atomic dispersion of supported noble metal nanoparticles
Source: Nat Commun. 2019 Nov 21;10:5281. doi: 10.1038/s41467-019-12965-1 (PMC6872874; doi:10.1038/s41467-019-12965-1)
Supplement: Supplementary file 1 — Supplementary Information [file 41467_2019_12965_MOESM1_ESM.docx]

**In Situ Formation of Mononuclear Complexes by Reaction-Induced Atomic Dispersion of Supported Moble Metal Nanoparticles**

**Feng *et al.***

**Table of Content**

**Supplementary Figures**

1. XRD patterns of samples with different treatment conditions 3

2. Rh atomic EDS mapping of Rh_1_/AC 4

3. *k*^2^ χ(*k*) oscillations of Rh/AC and Rh_1_/AC 5

4. The wavelet transform contour plots of Rh/AC and Rh_1_/AC 6

5. TPD spectra of Rh_1_/AC cooled by CO 7

6. The ATR-FTIR patterns of Rh_1_/AC 8

7. XPS spectra of Rh/AC and Rh_1_/AC 9

8. LDI/TOF MS for Rh_1_/AC 10

9. Time resolution EXAFS patterns of Rh/AC dispersion 11

10. Time resolution XRD patterns of Rh/AC dispersion 12

11. Temperature effect on Rh/AC dispersion 13

12. CO and CH_3_I effect on Rh/AC dispersion 14

13. MAS NMR of AC and that treated with H_2_ at 1273 K 15

14. The TPD-MS profiles of AC and the AC treated by H_2_ 16

15 The TEM images of the Rh/AC catalyst supported on AC 17

16 The model of support activated carbon (AC) for DFT calculation 18

17 DFT calculation for Rh/AC dispersion 19

18 Energy transformation of Rh(CO)_x_I_y_(O-AC) 20

19 NPs dispersed to single atom of Ru 21

20 NPs dispersed to single atom of Pd 22

21 NPs dispersed to single atom of Ag 23

**Supplementary Tables**

1. EXAFS quantitative analyses of Rh/AC and Rh_1_/AC 24

2. XPS data of Rh_1_/AC treated and cooled in CO or N_2_ atmosphere 25

3. ICP-OES measurement of Rh/AC and Rh_1_/AC 26

4. Time resolution EXAFS Quantitative analyses of Rh/AC dispersion 27

5. The structure parameters transformation of Rh/AC dispersion 28

6. EXAFS quantitative analyses of Ir_1_/AC and Pt_1_/AC 29





**Supplementary Figure 1 ǀ** XRD patterns of Rh/AC with different treatment conditions.


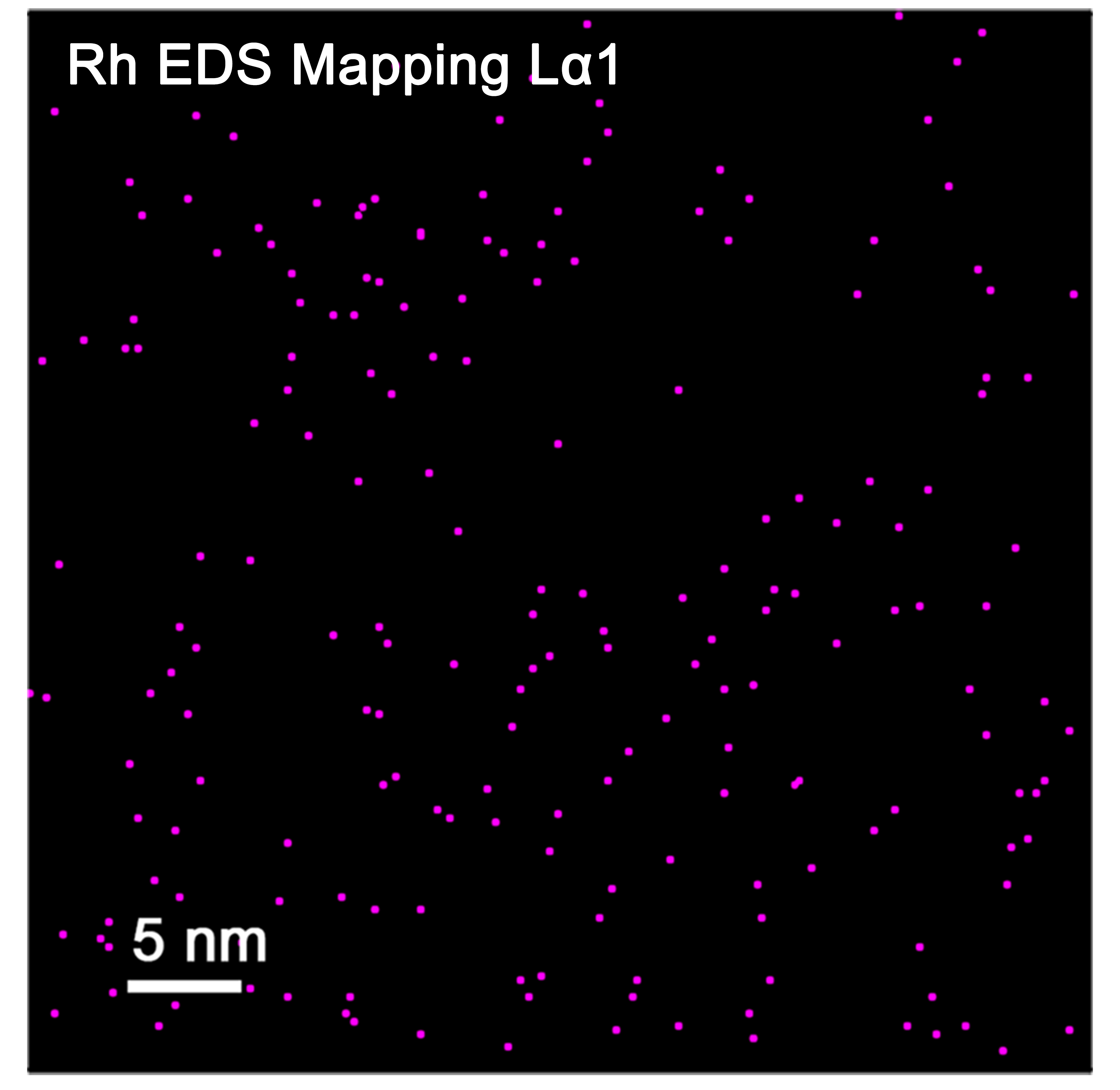


**Supplementary Figure 2 ǀ** Rh atomic EDS mapping of Rh_1_/AC.

**

**

**Supplementary Figure 3 ǀ** *k*^2^ χ(*k*) oscillations of Rh/AC and Rh_1_/AC.


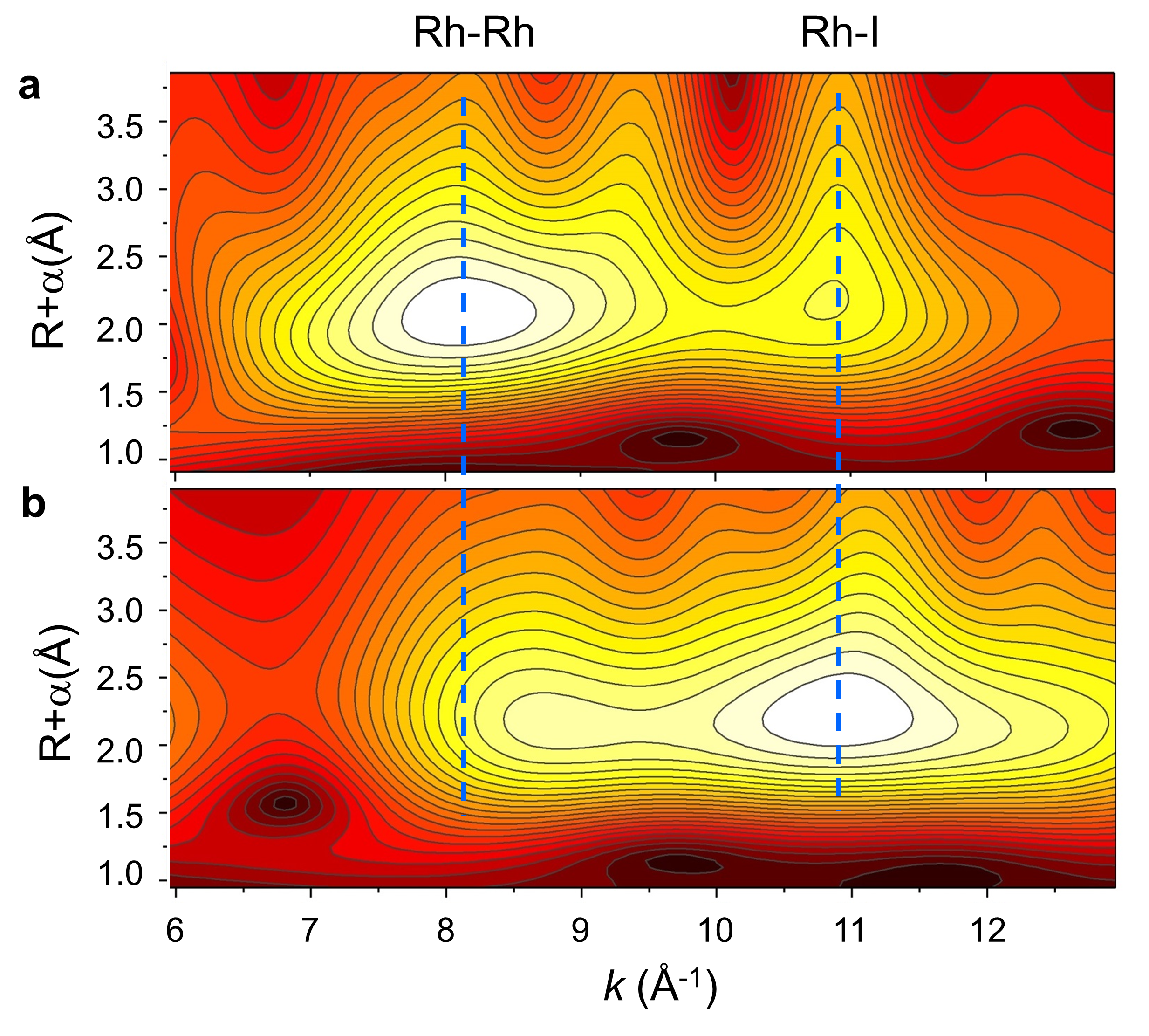


**Supplementary Figure 4 ǀ** The wavelet transform contour plots of *k*^2^-weighted χ(*k*) EXAFS signals of (**a**) Rh/AC and (**b**) Rh_1_/AC.

**

**

**Supplementary Figure 5 ǀ** CO-TPD spectra of Rh_1_/AC cooled by CO.

**

**

**Supplementary Figure 6 ǀ** ATR-FTIR patterns of Rh_1_/AC cooled in CO and N_2_. The peak at 2160 cm^-1^ is attributed to [Rh(CO)I_4_] species and the peaks at 2030 and 2017 cm^-1^ are belonged to [Rh(CO)_2_I_3_] species.





**Supplementary Figure 7 ǀ** XPS spectra of Rh/AC and Rh_1_/AC samples cooled in CO or N_2_.





**Supplementary Figure 8 ǀ** LDI/TOF-MS experiment for Rh_1_/AC. The signal of CO (m/z=28), Rh (m/z=127), and I • (m/z=127) were detected, indicating existence of CO and I • species in Rh_1_/AC.

**

**

**Supplementary Figure 9 ǀ** Time resolution EXAFS patterns of Rh/AC dispersion that treated with CO/CH_3_I at 513 K.





**Supplementary Figure 10 ǀ** Time resolution XRD patterns of Rh/AC dispersion that treated with CO/CH_3_I at 513K for different times.

**
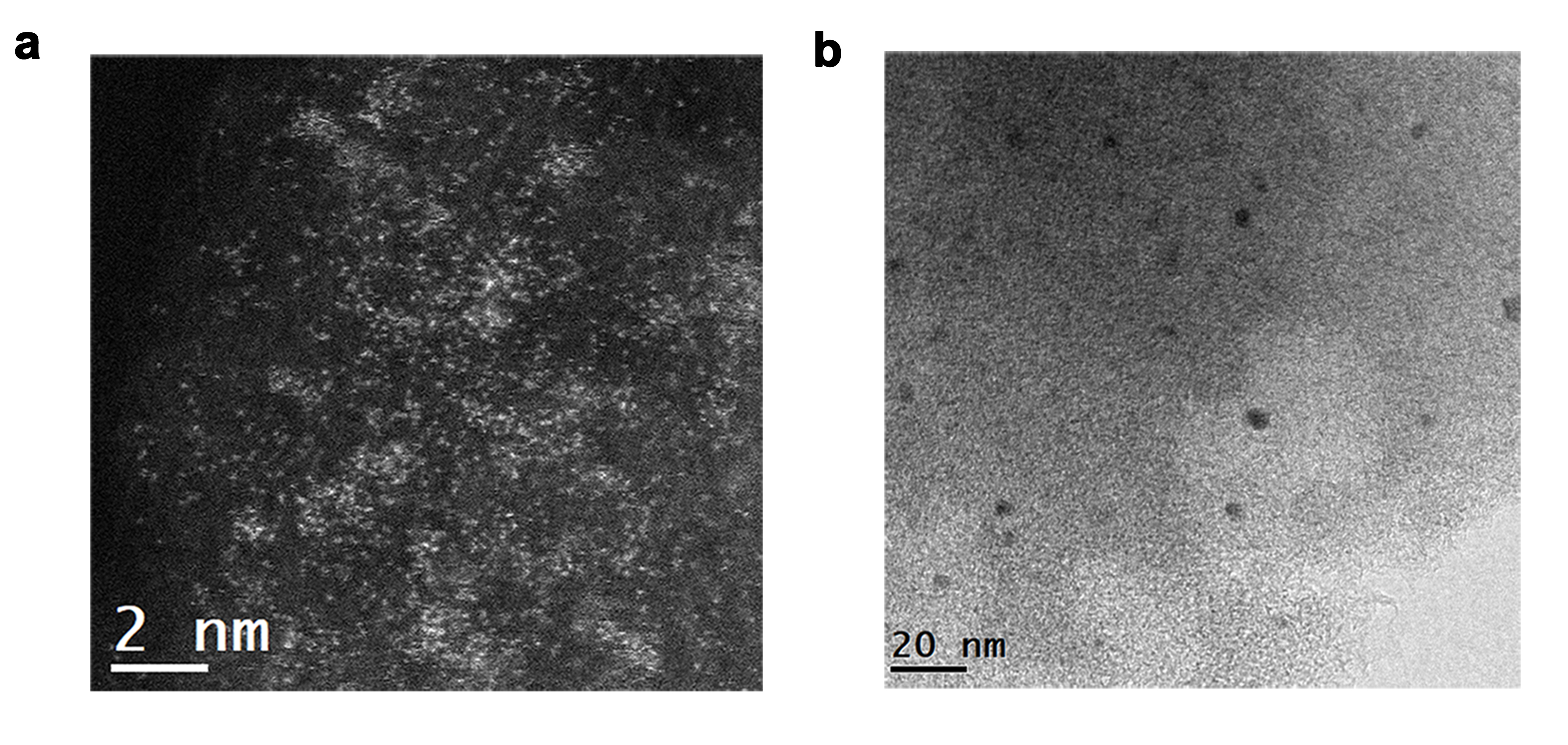
**

**Supplementary Figure 11 ǀ** Temperature effect on Rh/AC dispersion. HADDF-STEM images of (**a**) Rh/AC treated by CO/CH_3_I at 433 K for 6 h and (**b**) Rh/AC treated by CO/CH_3_I at 373 K for 6 h.

**
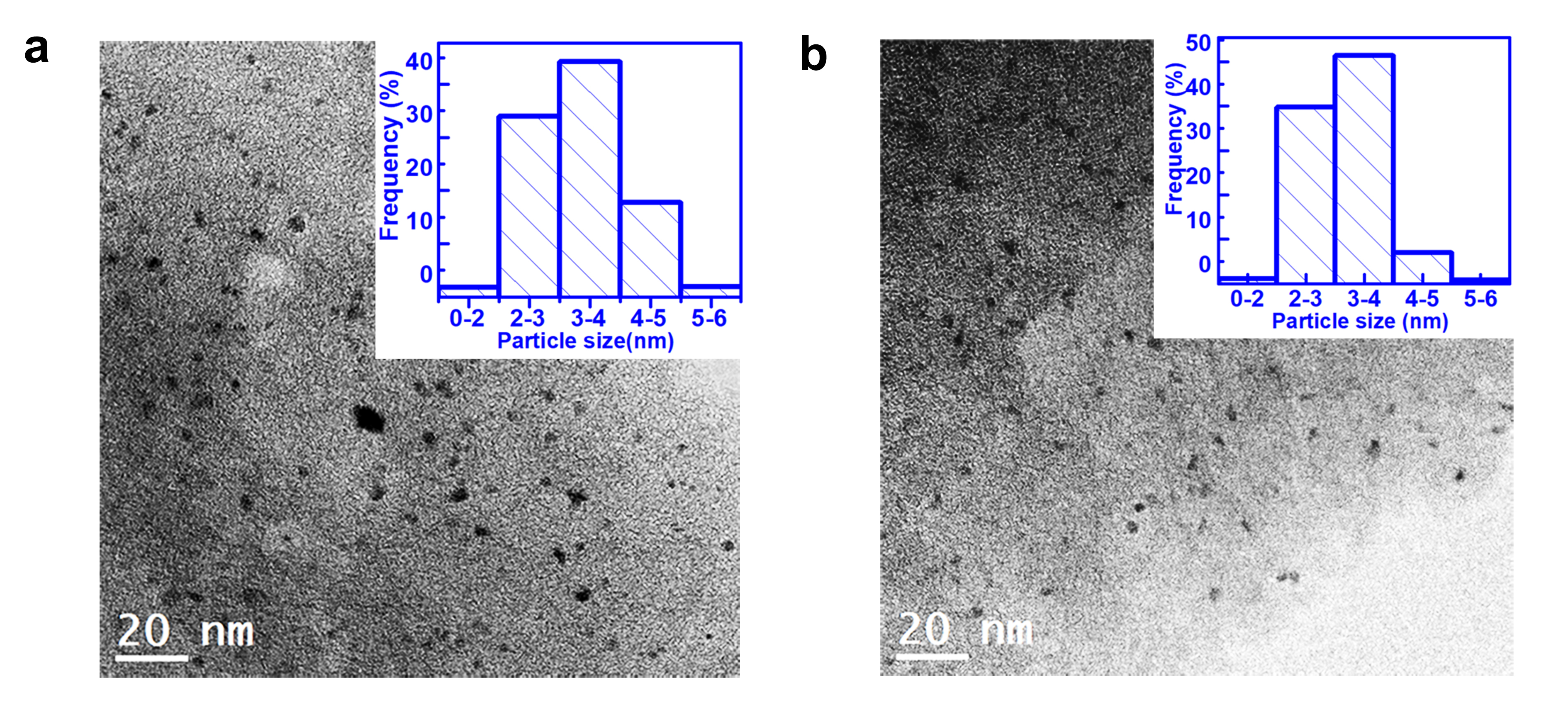
**

**Supplementary Figure 12 ǀ** CO and CH_3_I individual effect on Rh/AC dispersion. The HRTEM picture of Rh/AC treated by (**a**) CO and (**b)** by N_2_/CH_3_I at 513 K for 6 h, as well as their corresponding particle size distribution.

**

**

**Supplementary Figure 13 ǀ** MAS NMR spectra of AC and that treated with H_2_ at 1273 K for 6 h.


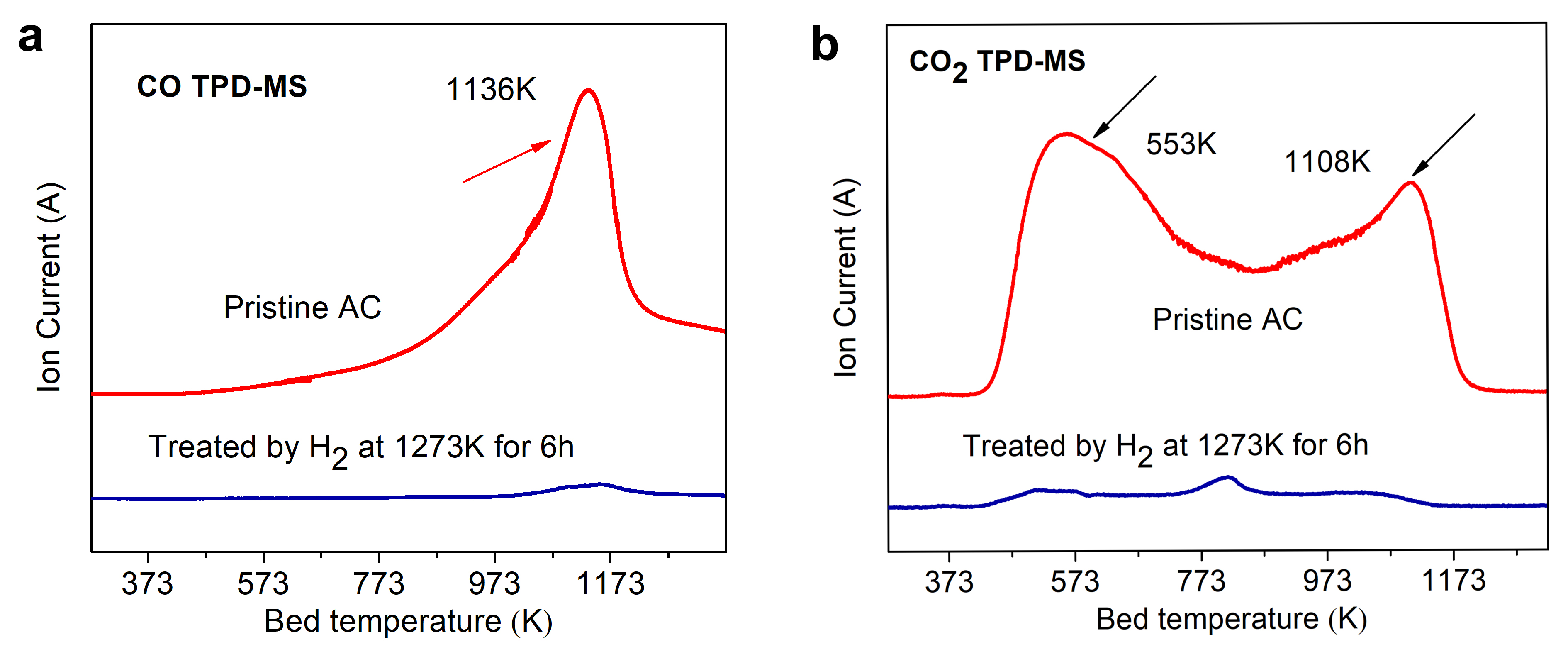


**Supplementary Figure 14 ǀ** The TPD-MS profiles of AC and the AC treated by H_2._ (**a**) CO, (**b**) CO_2_.

**
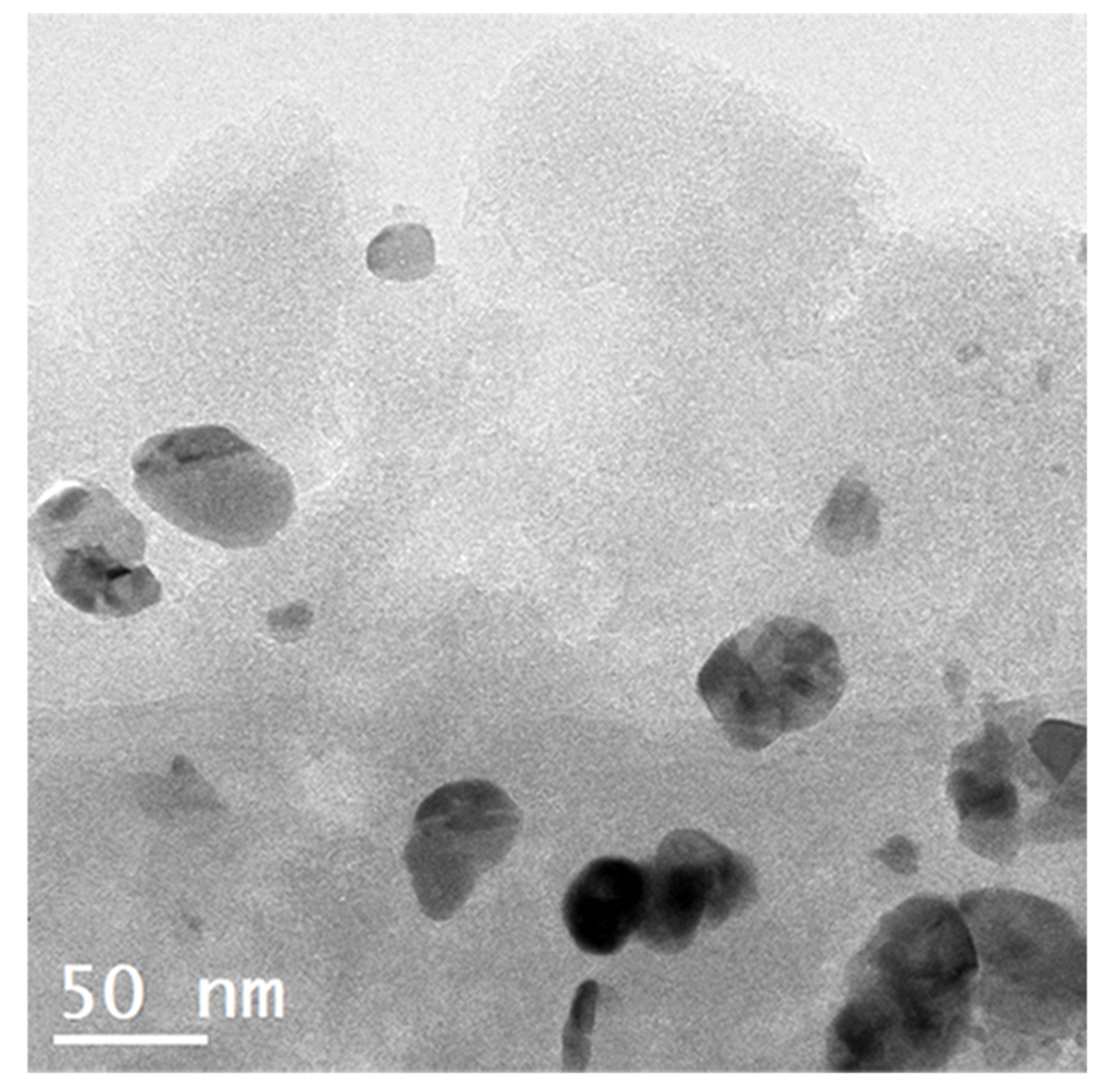
**

**Supplementary Figure 15 ǀ** The TEM images of the Rh/AC catalyst supported on AC. The AC was treated in a flow of H_2_ at 1273K for 6 h, then the Rh/AC was treated with CO/CH_3_I at 513 K for 6 h.

**
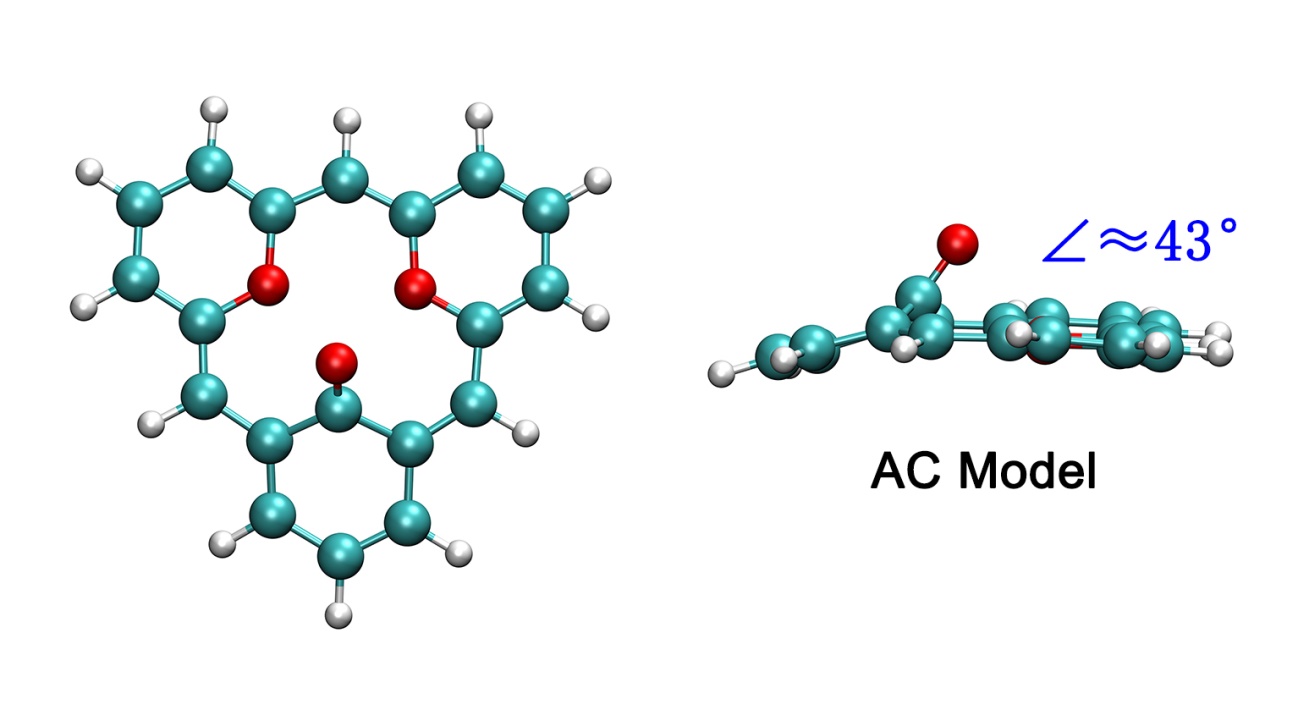
**

**Supplementary Figure 16 ǀ** The model of support activated carbon (AC) for DFT calculation.

**
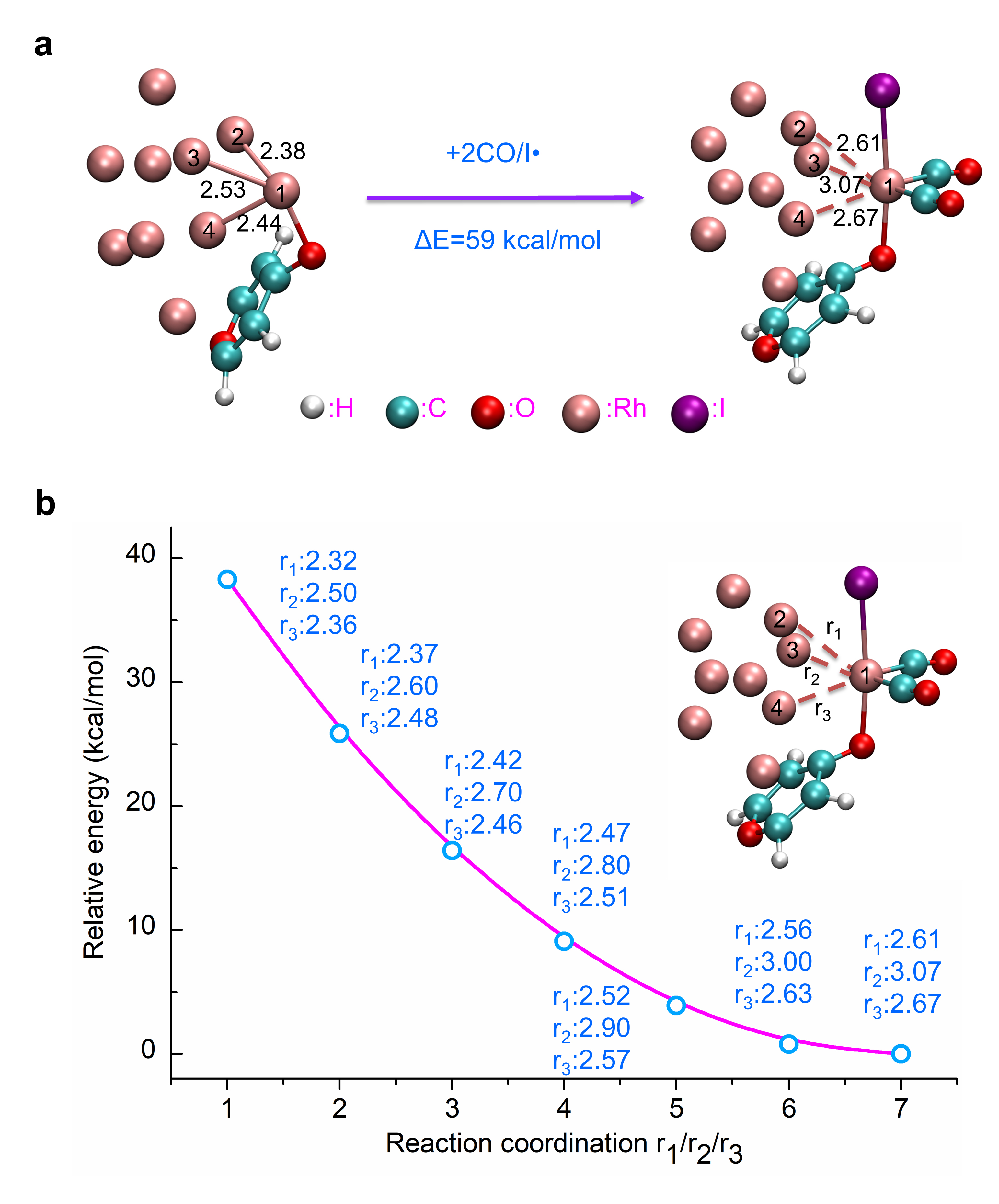
**

**Supplementary Figure 17 ǀ** DFT calculation for Rh/AC dispersion. (**a**) The dispersion model of Rh/AC attacked by CO and I•, as well as the system energy transformation of Rh NPs via one-by-one mechanism in **Figure. 3c,** (**b**) The potential energy curve of Rh(CO)_2_I(O-AC)/Rh NPs complex with selected r1/r2/r3 Rh-Rh bond. The blue data are the bond lengths of r1, r2 and r3, respectively.

**
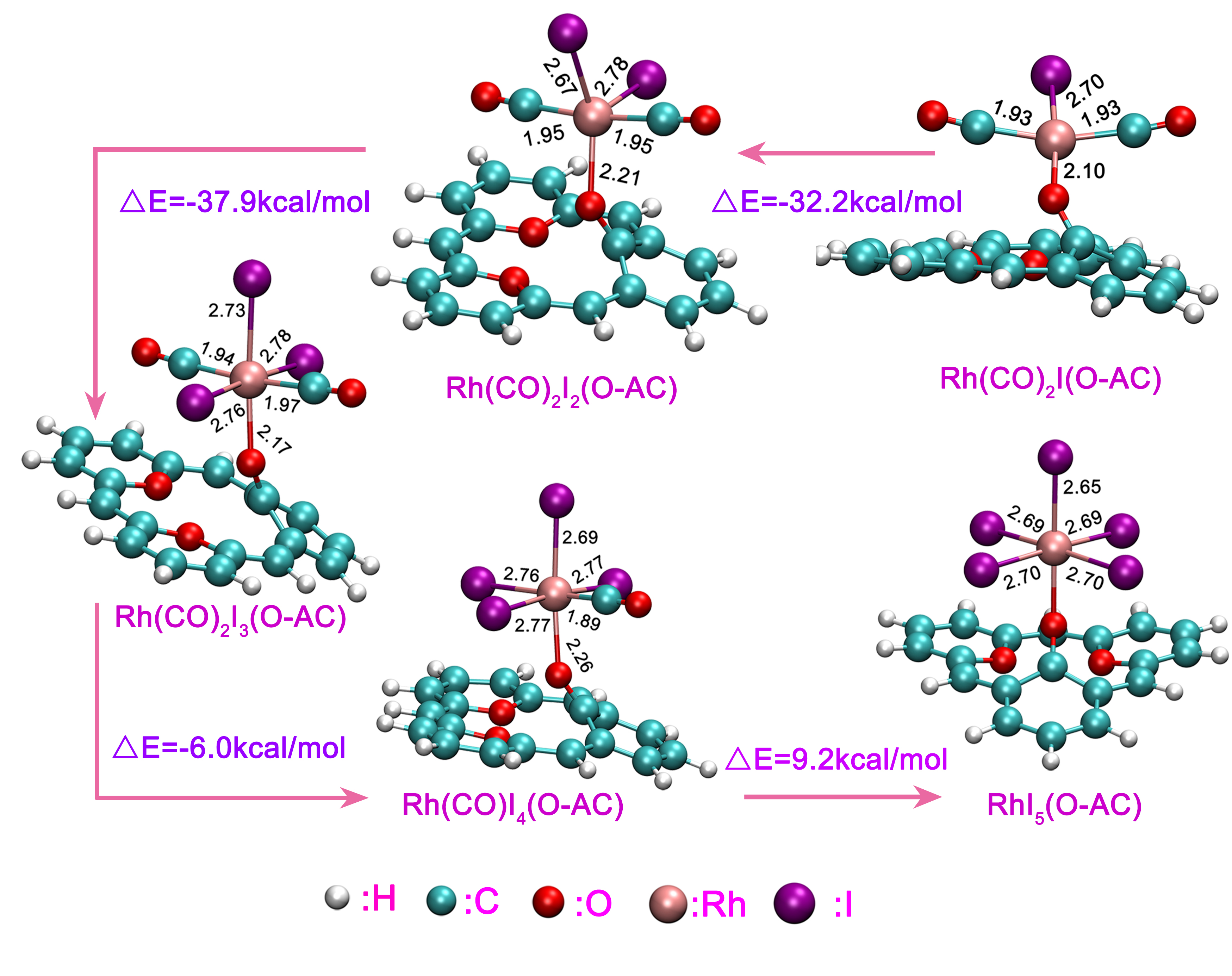
**

**Supplementary Figure 18 ǀ** Energy transformation of Rh(CO)_x_I_y_(O-AC). The model and energy transformation of the stable formation of Rh(CO)_2_I_3_(O-AC) and Rh(CO)I_4_(O-AC) complex corresponding to **Figure 3c**.

**
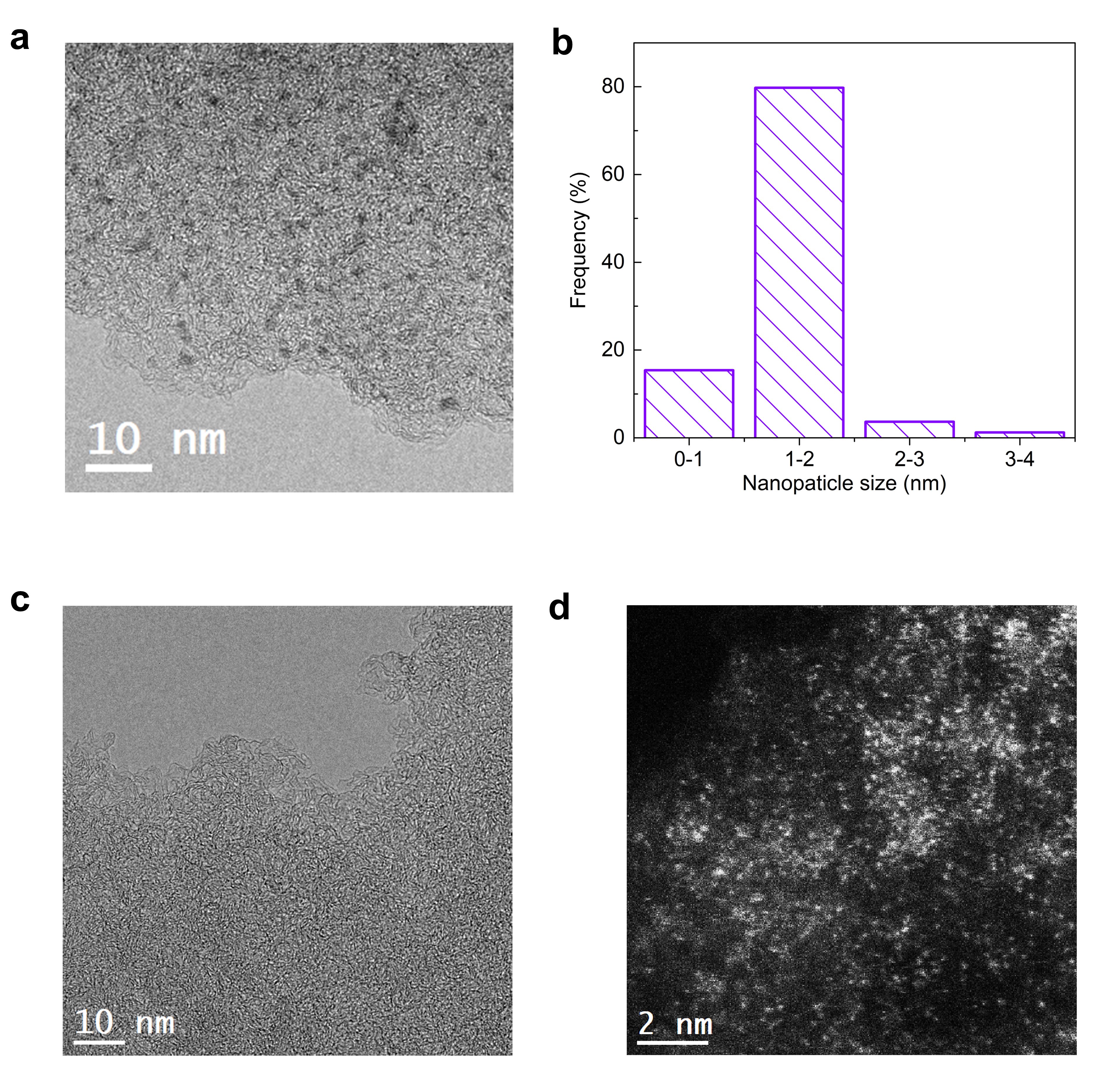
**

**Supplementary Figure 19 ǀ** NPs dispersed to single atom of Ru. (**a**) HRTEM picture and (**b**) the corresponding particle size distribution of Ru/AC, (**c**) HRTEM and (**d**) HAADF-STEM pictures of Ru_1_/AC.

**
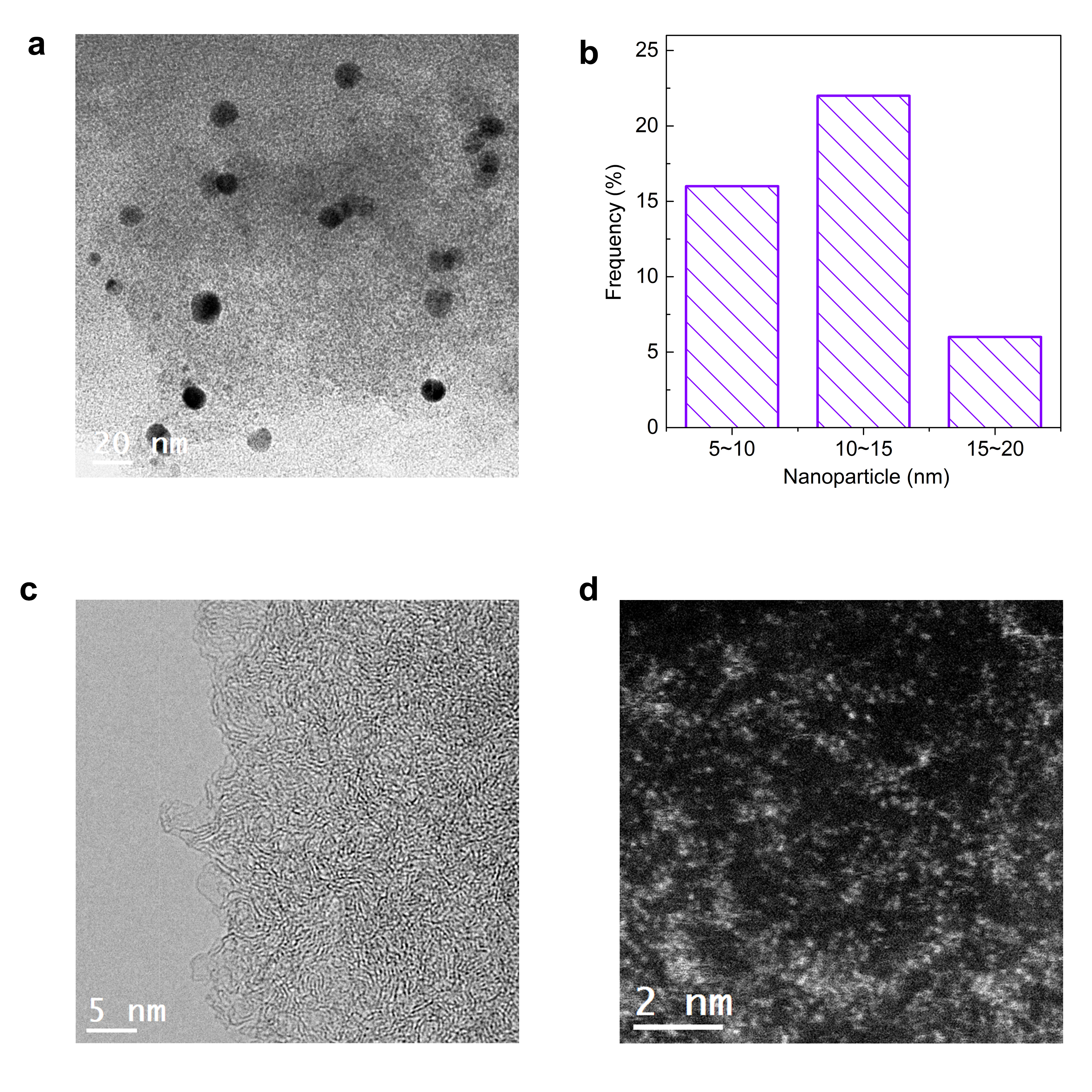
**

**Supplementary Figure 20** **ǀ** NPs dispersed to single atom of Pd. (**a**) HRTEM picture and (**b**) the corresponding particle size distribution of Pd/AC, (**c**) HRTEM and (**d**) HAADF-STEM pictures of Pd_1_/AC.

**
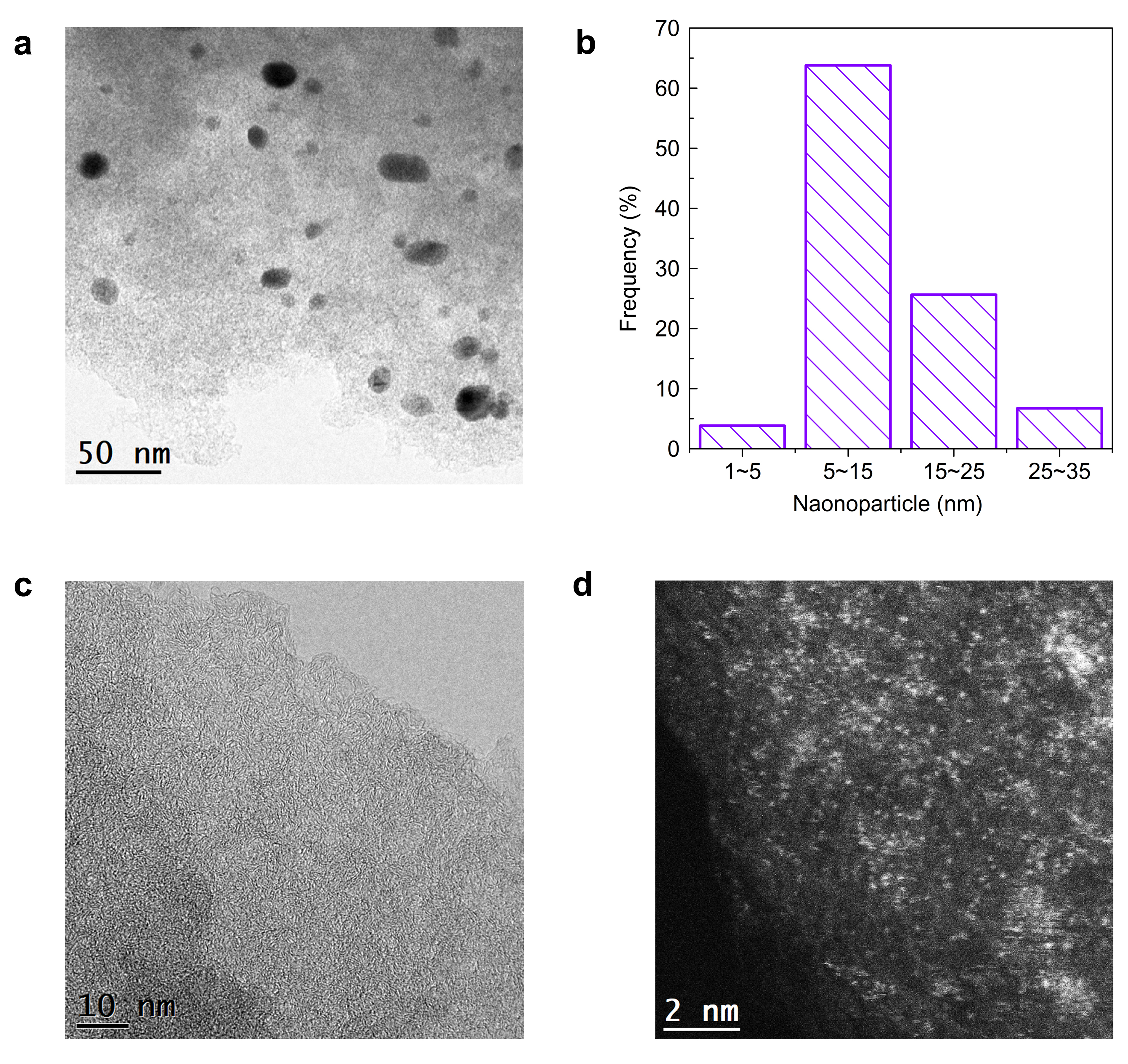
**

**Supplementary Figure 21 ǀ** NPs dispersed to single atom of Ag. (**a**) HRTEM picture and (**b**) the corresponding particle size distribution of Ag/AC, (**c**) HRTEM and (**d**) HADDF-STEM pictures of Ag_1_/AC.

**Supplementary Table 1 ǀ** EXAFS quantitative analyses of Rh/AC and Rh_1_/AC. Quantitative analyses of Rh-Rh, Rh-O, Rh-C and Rh-I contributions as measured by EXAFS in **Figure 1d**

| Samples | Shell | N | R (Å) | E_0_ | σ x 10^-3^ | R factor |
| --- | --- | --- | --- | --- | --- | --- |
| Rh/AC | Rh-O | 2.1 | 2.05 | -4.8 | 4.9 | 0.006 |
|  | Rh-Rh | 5.0 | 2.69 | -8.4 | 3.9 |  |
| Rh_1_/AC | Rh-CO | 1.7 | 1.88 | -5.2 | 7.9 | 0.006 |
|  | Rh-O | 1.0 | 2.09 | -10.0 | 4.6 |  |
|  | Rh-I | 3.7 | 2.67 | 1.4 | 3.1 |  |

**Supplementary Table 2 ǀ** XPS data of Rh_1_/AC cooled in CO or N_2_ atmosphere

| Samples | Rh 3d_5/2_ | | |
| --- | --- | --- | --- |
|  | B. E. (eV) | Area (%) | Assign |
| Rh_1_/AC  cooled in CO | 308.5 | 81.5 | Rh^3+^ |
|  | 310.8 | 19.5 | -- |
| Rh_1_/AC  cooled in N_2_ | 308.2 | 63.3 | Rh^3+^ |
|  | 309.5 | 36.7 | -- |

**Supplementary Table 3 ǀ** ICP-OES measurement of Rh/AC and Rh_1_/AC

| ICP-OES | Content (wt. %) |
| --- | --- |
| Rh/AC | 4.81 |
| Rh_1_/AC | 4.79 |

**Supplementary Table 4 ǀ** Time resolution EXAFS quantitative analyses of Rh/AC dispersion

| Samples | Shell | N | R (Å) | E_0_ | σ x 10^-3^ | R factor |
| --- | --- | --- | --- | --- | --- | --- |
| 0 min | Rh-O | 2.1 | 2.05 | -4.8 | 4.9 | 0.006 |
|  | Rh-Rh | 5.0 | 2.69 | -8.4 | 3.9 |  |
| 2 min | Rh-CO | 1.4 | 1.89 | 0.9 | 3.8 | 0.004 |
|  | Rh-O | 1.0 | 2.06 | -4.3 | 3.0 |  |
|  | Rh-I | 3.3 | 2.66 | 0.7 | 3.1 |  |
|  | Rh-Rh | 0.7 | 2.68 | 9.8 | 5.2 |  |
| 5 min | Rh-CO | 2.2 | 1.84 | -9.0 | 7.0 | 0.008 |
|  | Rh-O | 1.2 | 2.09 | -5.1 | 7.1 |  |
|  | Rh-I | 3.3 | 2.67 | 1.9 | 3.0 |  |
|  | Rh-Rh | ＜0.3 |  |  |  |  |
| 15 min | Rh-CO | 1.5 | 1.86 | -9.1 | 3.5 | 0.009 |
|  | Rh-O | 1.0 | 2.09 | -9.9 | 3.1 |  |
|  | Rh-I | 3.6 | 2.67 | 1.5 | 3.2 |  |
| 1 h | Rh-CO | 2.3 | 1.89 | -4.0 | 7.0 | 0.006 |
|  | Rh-O | 1.2 | 2.09 | -9.3 | 4.5 |  |
|  | Rh-I | 3.6 | 2.67 | 1.3 | 3.1 |  |
| 6 h | Rh-CO | 1.7 | 1.88 | -5.2 | 7.9 | 0.006 |
|  | Rh-O | 1.0 | 2.09 | -10.0 | 4.6 |  |
|  | Rh-I | 3.7 | 2.67 | 1.4 | 3.1 |  |

**Supplementary Table 5 ǀ** The structure parameters of Rh/AC dispersion. The AC supported Rh NPs complex which Rh_1_ atom is attacked with and without CO/I•

| Rh NPs/(O-AC)  Complex | Bond Distance  (Å) | Rh NPs/(O/AC)/(CO)_2_/I•  Complex | Bond Distance  (Å) |
| --- | --- | --- | --- |
| Rh1-Rh2 | 2.38 | Rh1-Rh2 | 2.61 |
| Rh1-Rh3 | 2.53 | Rh1-Rh3 | 3.07 |
| Rh1-Rh4 | 2.44 | Rh1-Rh4 | 2.67 |
| Rh2-Rh3 | 2.47 | Rh2-Rh3 | 2.62 |
| Rh2-Rh4 | 2.61 | Rh2-Rh4 | 2.52 |
| Rh2-Rh9 | 2.49 | Rh2-Rh8 | 2.51 |
| Rh2-Rh10 | 2.46 | Rh2-Rh10 | 2.46 |
| Rh3-Rh4 | 2.73 | Rh3-Rh4 | 2.54 |
| Rh3-Rh6 | 2.48 | Rh3-Rh7 | 2.54 |
| Rh3-Rh8 | 2.47 | Rh3-Rh9 | 2.45 |
| Rh3-Rh10 | 2.60 | Rh3-Rh10 | 2.50 |
| Rh4-Rh5 | 2.47 | Rh4-Rh5 | 2.48 |
| Rh4-Rh6 | 2.56 | Rh4-Rh6 | 2.52 |
| Rh4-Rh7 | 2.50 | Rh4-Rh7 | 2.59 |
| Rh4-Rh9 | 2.67 | Rh4-Rh8 | 2.50 |
| Rh5-Rh6 | 2.53 | Rh5-Rh6 | 2.51 |
| Rh5-Rh7 | 2.59 | Rh5-Rh7 | 2.69 |
| Rh6-Rh7 | 2.51 | Rh6-Rh7 | 2.49 |
| Rh6-Rh8 | 2.54 | Rh6-Rh8 | 2.56 |
| Rh7-Rh8 | 2.53 | Rh6-Rh9 | 2.52 |
| Rh7-Rh9 | 2.55 | Rh7-Rh9 | 2.52 |
| Rh8-Rh9 | 2.42 | Rh8-Rh9 | 2.48 |
| Rh8-Rh10 | 2.52 | Rh8-Rh10 | 2.48 |
| Rh9-Rh10 | 2.49 | Rh9-Rh10 | 2.54 |

**Supplementary Table 6 ǀ** EXAFS quantitative analyses of Ir_1_/AC and Pt_1_/AC. Quantitative analyses of M-M, M-O, M-CO and M-I contributions as measured by EXAFS (**Figure 5c** and **5f**)

| Samples | Shell | N | R (Å) | E_0_ | σ x 10^-3^ | R factor |
| --- | --- | --- | --- | --- | --- | --- |
| Pt_1_/AC | Pt-CO | 1.2 | 1.84 | -9.0 | 7.0 | 0.006 |
|  | Pt-O | 1.0 | 2.11 | 9.0 | 8.5 |  |
|  | Pt-I | 3.3 | 2.60 | 8.6 | 3.0 |  |
| Ir_1_/AC | Ir-CO | 2.0 | 1.80 | 7.2 | 8.7 | 0.019 |
|  | Ir-O | 1.0 | 1.98 | 8.0 | 3.6 |  |
|  | Ir-I | 3.0 | 2.68 | 3.0 | 10.7 |  |
