# Supplementary material for: In situ formation of mononuclear complexes by reaction-induced atomic dispersion of supported noble metal nanoparticles
Source: Nat Commun. 2019 Nov 21;10:5281. doi: 10.1038/s41467-019-12965-1 (PMC6872874; doi:10.1038/s41467-019-12965-1)
Supplement: Supplementary file 2 — Peer Review [file 41467_2019_12965_MOESM2_ESM.pdf]

Reviewers' comments:

Reviewer #1 (Remarks to the Author):

The authors demonstrate that activated carbon-supported Rh nanoparticles (Rh/AC) disperse upon simultaneous reaction with carbon monoxide and methyl iodide to form isolated molecular Rh species that are catalytically active and stable for methanol carbonylation. Crucially, this dispersion was only observed in the presence of the native surface oxygen on activated carbon, as evidenced by formation of ~50 nm Rh particles on activated carbon pre-reduced at 1273 K. The authors elucidated this transformation by applying a suite of ex situ and in situ techniques—including TEM, XRD, XAS, TPD, ATR-FTIR, XPS, and EPR spectroscopy—to characterize materials derived from Rh/AC after various chemical treatments. Coordination numbers from EXAFS, together with FTIR, EPR, and XPS spectra, indicate that the mononuclear Rh species co-exist as octahedrally coordinated Rh(III) and Rh(IV) species, namely Rh(CO)2I3(O-AC) and Rh(CO)I4(O-AC). Moreover, HAADF-STEM images and EXAFS spectra after various CO/CH3I exposure times point towards a gradual Rh dispersion process, which is purported to be an atom-by-atom removal on the basis of DFT calculations. Finally, the authors show that the atomically dispersed, immobilized Rh complexes are active methanol carbonylation catalysts, exhibiting a stable turnover frequency three times higher than that of the analogous unsupported molecular complex, even in the presence of hydrogen sulfide.

This manuscript presents comprehensive characterization of the dynamism of a heterogeneous metal catalyst during reaction, and highlights a distinctly novel role of the activated carbon support as an essential chemical participant in forming the catalytically active site for carbonylation. However, despite their thorough materials characterization, the authors do not substantively compare the reaction kinetics of the heterogeneous and homogeneous catalysts. Additionally, the article is weakened by the authors' grand claims and anecdotes of practical viability, which are unconvincing without any techno-economic comparison with the Monsanto and Cativa processes. Still, the fundamental chemical insights provided by this work regarding the spontaneous transformation of metal nanoparticles into molecular catalysts are alone sufficiently novel and pertinent to a wide community of chemists and engineers now working at the intersection of heterogeneous and homogeneous catalysis. I recommend publication pending major revisions.

Major comments:
• Without any mention of the limiting reagent conversion, the authors present carbonylation rates and selectivities and claim that the monodisperse Rh1/AC catalyst is three times more active than the homogeneous catalyst. It is impossible to interpret the reported rates and selectivities without knowing the conversions at which they were measured. All rates should be reported at differential conversion to reflect the kinetic regime, and selectivities must be reported at similar conversions when multiple reactions occur in series (as is the case here).
• If the Rh1/AC catalyst really is intrinsically more active than the homogeneous catalyst, reaction kinetics experiments would buttress and even supersede DFT calculations for rationalizing this observation. Thus, we recommend reaction kinetics measurements to compare the apparent activation energies and reaction orders in CO, CH3OH, and CH3I of the heterogeneous Rh1/AC and homogeneous Rh(CO)2I2 catalysts. Based on the DFT-informed hypothesis of the authors, the heterogeneous catalyst would exhibit a lower apparent activation energy than would the homogeneous catalyst. Comparison of reaction orders would provide insight into any possible mechanistic differences between the heterogeneous and homogeneous catalysts.
• In Figure 5d, please also present a TOF for the homogeneous catalyst in the presence of H2S for comparison with the monodisperse Rh1/AC tolerance. This is important for the reader to assess the relative tolerance of the monodisperse Rh1/AC and homogeneous catalysts.
• The authors mention acetyl selectivities as low as 20%, but make no mention of the other products. The species accounting for the remaining carbon must, if possible, be enumerated and quantified. If this is not possible, then discussion of the analytical limitations is warranted. It is also essential to mention how selectivity was calculated (i.e., was it calculated based on reactant conversion or total

product formation).
• The authors attribute the enhanced activity of the heterogenized Rh complex (relative to the homogeneous analog) to the AC-O ligand, with support from DFT calculations. Have the authors considered or analyzed the implications of band spreading at the Rh center upon electronic coupling to the band structure of the graphitic activated carbon surface?
• Please provide all specific chemicals and suppliers used in the methods section. This is essential for reproducibility.
• Presumably, hydroiodic acid was present from the conversion of acetyl iodide to methyl acetate and acetic acid. How were the liquid products worked up prior to GC injection to remove or handle HI? This is also essential for reproducibility.
• Even in ultra-high purity CO, iron pentacarbonyl is a common impurity formed upon reaction with steel cylinders during storage. Was care taken to remove Fe(CO)5 from the CO stream? Carbonyl complexes are known promoters of the Cativa Ir-based catalyst, so it's conceivable that Fe(CO)5 might have some effect on the Rh system. At the very least it is worth mentioning whether or not the CO stream was purified for the purposes of reproducibility.
• We recommend that the authors either remove discussion of industrial viability, or present a fell techno-economic analysis—comparing their process to the Monsanto and Cativa processes—to support their claims about applicability.
• In the EXAFS data, the Rh-Rh and Rh-I features appear essentially identical in R-space. Is it possible to reliably deconvolute these features in the fitting process, especially during the transient studies?

Minor comments:
• The sentence structure and grammar often distract from the science trying to be conveyed. As such, the manuscript (including its title) would substantially benefit from thorough grammatical and syntactical revisions to improve clarity.

Reviewer #2 (Remarks to the Author):

This manuscript presents a study of a single metal site heterogeneous Rh catalyst for methanol carbonylation that form in situ upon exposure of Rh nanoparticles to a CH3I/CO environment. Much experimental and theoretical effort was spent in identifying the structure of the single Rh atom complexes, which are shown to be much more active than their nanoparticle and homogeneous counterparts. While the results here are interesting, I do not feel that they warrant publication in Nature Communications for several reasons. First, the authors present the idea of using heterogeneous mononuclear complexes as substitutes for nanoparticle catalysts as a relatively new idea. However, researchers such as Bruce Gates have been studying such complexes for decades. As such, I do not feel that the authors have done a thorough review of the literature related to supported single metal complexes. Second, this method of single atom complex stabilization with halohydrocarbons is not likely to be extendable to other catalytic reactions as methanol carbonylation actively requires and uses I ligands around the metal complex center, making this particular reaction the most ideal or perfect candidate reaction, whereas such I ligands can have negative consequences on other reactions. Third, I have several major critiques of the theoretical methods and design of the models used here (see below for details), which is used here to rationalize the structure of the catalyst and the improved performance of the single atom Rh complexes. As such, I do not recommend this manuscript for publication in Nature Communications.

Theory Critiques
- Besides a brief comparison in turnover frequencies and some elementary barriers, no significant comparison is made between the homogeneous Rh catalyst standard for methanol carbonylation and the HSMSCs here. No arguments are presented here why this heterogeneous system outperforms the homogeneous catalyst. What structural or charge effects from the surface immobilization lead to decreased elementary barriers and increased turnover frequencies? The inclusion of theoretical models

allows for deeper understanding of the active site's structural and electronic relation to performance, and the lack of such analysis weakens this manuscript.

- The choice of Rh HSMSC structure for modeling the methanol carbonylation reaction pathway is questionable. First, the authors find the Rh HSMSCs exists as +3 and +4 complexes and propose structures [Rh(CO)2I3(O-AC) and Rh(CO)I4(O-AC)], with both charge states having 6-fold coordination. However, in Figure 5 the active complex is modeled to be Rh(CO)2I2(O-AC) with Rh having a charge of +2, with the +4 complex forming only after CH3I oxidative addition. Under the characterization conditions, the Rh complex was exposed to the CH3I/CO environment required for reaction to occur, so on average the characterizations should capture the dominant surface species and the theory in Figure 5 suggests this to be Rh(CH3CO)(CO)I3(O-AC), observable with in situ IR spectroscopy, where Rh is said to be +4. How does the theory justify such models in relation to the detailed experimental characterizations performed on the system? Furthermore, the proposed mechanism shows that the Rh requires transitions between +2, a rare Rh complex state, and +4. What is the role and function of the +3 Rh complex in this system? Homogeneously, the Rh complex cycles between the +1 and +3 charge states. What is the justification for the models with +2/+4 transitions for this system?
- As this work is concerned with the in situ formation of catalytically active sites, the theoretical results here should be presented as free energies under reaction/treatment conditions. For example, under reaction conditions, what is the dominant surface complex from those presented in Figure 3? The adsorption of CO and I will see significant entropy effects due to loss of translational and rotational degrees of freedom that could significantly affect the stability of each complex and such factors are currently unaccounted for. A similar question can be asked of the effect of entropic losses on the barriers shown in Figure 5.
- The argument against possible dimer Rh structures is made primarily from the weak interaction found to occur between two fully coordinated Rh complexes (Figure S7). However, this is measured entirely by the Rh-Rh distance in the optimized structures. What is the interaction energy between these complexes? Why were no dimer Rh complexes with I ligands bridging the metal centers tested? More work is needed in regard to fully defining the single atom nature of the complexes.
- The formation of Rh single atom complexes from Rh nanoparticles shown in Figure S16 suggests that the O-AC site is a critical component required to form the Rh complexes. Is this true? I agree that the O-AC are likely sites for the stabilization of such isolated species, but are they required to directly participate in the transition state for breaking off at Rh atom? Furthermore, we are shown that the formation of such Rh complexes is highly favorable in Figure S16, but this does not include entropy loss for the adsorption of CO and I, as discussed above.
- The model of the O-AC is not very well justified. Only a single oxygen functionality is tested as a possible site for stabilizing the Rh complexes, but many different oxygen functionalities exist on activated carbon. What is the rationale for this choice of functionality? What are the effects of the oxygen functionality on the stability and activity of the complex? Furthermore, the model surface itself is small, comprising only 6 ring structures. How was such a model chosen? What is the effect of surface size on the calculated energetics?
- A major argument made by the authors is that these Rh complexes are good catalysts because of their resistance to H2S poisoning, which is offhandedly argued to be due to no opportunity for H2S activation from fast oxidative addition. However, this argument should be improved by using the theoretical models to examine the activation of H2S on the Rh complexes and comparing the energy pathways directly and more quantitatively.
- Gaussian calculations used dispersion corrections, while no such corrections were included in the VASP calculations. What is the justification for not including dispersion corrections when modeling the interaction of the complex with a carbon surface fragment where such corrections are likely to be important?

General Critiques

- The writing in places is unpolished and casual (specifically in the introduction section). Examples can be seen in line 2 and 5 on page 3 and line 1 and 9 on page 4.
- Distinct lack of references to support key claims in the introduction. For example on page 3 second

paragraph, the authors discuss previous strategies for stabilizing HSMSCs in terms of surface/support modification and claim that these previous methods do not work under "practical conditions". However, no citations are provided to support this point. In fact, methods for modifying the surface to enable highly stable single metal sites to exist has been shown to be a viable method [Ref. 11 in the text; Science 2017, 358, 1419-1423; ACS Catal., 2019, 9, 1595-1604]. As a more minor example, on page 4 second paragraph no references are provided in relation to the Cativa and Monsanto processes.

- Figure 4a scheme is very confusing as it appears to be some type of reaction pathway, but is not a true reaction pathway as the attack by CO and I are presented in panels b-e as parallel possible reactions.
- Figure 5c should include high quality images of the model structures directly, instead of Lewis structure cartoons of the reactive structures.

Dear referees,

On behalf of the co-authors, we thank you very much for your comments. These comments are very useful to the improvement of our manuscript and the future work. We have carefully considered the reviewers' comments and tried our best to revise manuscript.

Sincerely yours,

Prof. Yunjie Ding (Ph. D)

Dalian Institute of Chemical Physics, Chinese Academy of Sciences

457 Zhongshan Road, Dalian 116023, P. R. China

Email: dyj@dicp.ac.cn

Response to the reviewers' comment

Reviewer#1

The authors demonstrate that activated carbon-supported Rh nanoparticles (Rh/AC) disperse upon simultaneous reaction with carbon monoxide and methyl iodide to form isolated molecular Rh species that are catalytically active and stable for methanol carbonylation. Crucially, this dispersion was only observed in the presence of the native surface oxygen on activated carbon, as evidenced by formation of ~50 nm Rh particles on activated carbon pre-reduced at 1273 K. The authors elucidated this transformation by applying a suite of ex situ and in situ techniques-including TEM, XRD, XAS, TPD, ATR-FTIR, XPS, and EPR spectroscopy-to characterize materials derived from Rh/AC after various chemical treatments. Coordination numbers from EXAFS, together with FTIR, EPR, and XPS spectra, indicate that the mononuclear Rh species co-exist as octahedrally coordinated Rh(III) and Rh(IV) species, namely $Rh(CO)_2I_3$(O-AC) and $Rh(CO)I_4$(O-AC). Moreover, HAADF-STEM images and EXAFS spectra after various CO/CH_3I exposure times point towards a gradual Rh dispersion process, which is purported to be an atom-by-atom removal on the basis of DFT calculations. Finally, the authors show that the atomically dispersed, immobilized Rh complexes are active methanol carbonylation catalysts, exhibiting a stable turnover frequency three times higher than that of the analogous unsupported molecular complex, even in the presence of hydrogen sulfide. This manuscript presents comprehensive characterization of the dynamism of a heterogeneous metal catalyst during reaction, and highlights a distinctly novel role of the activated carbon support as an essential chemical participant in forming the catalytically active site for carbonylation. However, despite their thorough materials characterization, the authors do not substantively compare the reaction kinetics of the heterogeneous and homogeneous catalysts. Additionally, the article is weakened by the authors' grand claims and anecdotes of practical viability, which are unconvincing without any techno-economic comparison with the Monsanto and Cativa processes. Still, the fundamental chemical insights provided by this work regarding the spontaneous

transformation of metal nanoparticles into molecular catalysts are alone sufficiently novel and pertinent to a wide community of chemists and engineers now working at the intersection of heterogeneous and homogeneous catalysis. I recommend publication pending major revisions.

Response: The spontaneous transformation of metal nanoparticles into molecular catalysts and its superior activity in heterogeneous methanol carbonylation are indeed the highlights of our work. Thanks very much for reviewer' appreciation and comments for our work. However, another referee raised doubts on the reaction mechanism of heterogeneous methanol carbonylation, especially the Rh complex cycle between +2 and +4 charge states. This is understandable. For the probe reaction of heterogeneous methanol, there are great difficulties to directly in-situ capture the dominant surface species on the activated carbon supported catalyst by infrared spectroscopy due to the fact that the transmitted or reflected infrared spectroscopy signals of the activated carbon are very poor in the in-situ cell. Without the full evidences of experimental data, the sole theoretical calculations to explain the reaction mechanism may be unconvincing and questionable. Therefore, in the revised manuscript, we deleted the discussion of heterogeneous carbonyaltion. Based on the comments of reviewers and the latest advances of our work, we focused the spontaneous transformation of metal nanoparticles into mononuclear complexes and the universality of the method for most noble metals. We will further investigate the reaction mechanism of heterogeneous methanol carbonylation according to the comments of referees and search evidential experimental data.

Comments: Without any mention of the limiting reagent conversion, the authors present carbonylation rates and selectivities and claim that the monodisperse Rh_1/AC catalyst is three times more active than the homogeneous catalyst. It is impossible to interpret the reported rates and selectivities without knowing the conversions at which they were measured. All rates should be reported at differential conversion to reflect the kinetic regime, and selectivities must be reported at similar conversions when multiple reactions occur in series (as is the case here).

Response: The selectivity for acetyl species (CH_3COOH and CH_3COOCH_3) was higher than 98% and the conversion of methanol was kept at 85%. The Rh NPs with ca. 2-5 nm supported on SiO_2 (Rh/SiO_2) with 1.0 wt.% loading (**Figure S24**) showed a poor activity of 10 mol_{acetyl}/mol_{Rh}/h and a lower selectivity of 20% to acetyl, only about one three-hundredth and one fifth of those for Rh_1/AC, respectively. The conversion of methanol on Rh/SiO_2 was lower than 1% and the selectivity to the CH_4 reached 80%. As stated above, the discussion about heterogeneous methanol carbonylation was deleted in the revised manuscript.

Comments: If the Rh_1/AC catalyst really is intrinsically more active than the homogeneous catalyst, reaction kinetics experiments would buttress and even supersede DFT calculations for rationalizing this observation. Thus, we recommend reaction kinetics measurements to compare the apparent activation energies and reaction orders in CO, CH_3OH, and CH_3I of the heterogeneous Rh_1/AC and homogeneous $Rh(CO)_2I_2$ catalysts. Based on the DFT-informed hypothesis of the authors, the heterogeneous catalyst would exhibit a lower apparent activation

energy than would the homogeneous catalyst. Comparison of reaction orders would provide insight into any possible mechanistic differences between the heterogeneous and homogeneous catalysts.

Response: The apparent activation energies on Rh_1/AC catalyst and homogeneous $[Rh(CO)_2I_2]^-$ catalyst were measured. The apparent activation energy (E_a) of the Rh_1/AC catalyst is 21.1 kcal/mol, obviously lower than 35.9 kcal/mol of the homogeneous $[Rh(CO)_2I_2]^-$ catalyst. This indicates the Rh_1/AC catalyst really is intrinsically more active than the homogeneous catalyst. Reaction orders in CO, CH_3OH, and CH_3I of the heterogeneous Rh_1/AC and homogeneous $Rh(CO)_2I_2$ catalysts are testing. As stated above, the discussion about heterogeneous methanol carbonylation was deleted in the revised manuscript. The comments are very helpful for the future work.

Comments: In Figure 5d, please also present a TOF for the homogeneous catalyst in the presence of H_2S for comparison with the monodisperse Rh_1/AC tolerance. This is important for the reader to assess the relative tolerance of the monodisperse Rh_1/AC and homogeneous catalysts.

Response: The suggestion was very useful to assess the relative tolerance of the monodisperse Rh_1/AC and homogeneous catalysts. We really conducted the experiment for the homogeneous catalyst in the presence of H_2S and its activity decrease dramatically. However, in the present state, it is difficult to understand the intrinsic reason for the sulfur tolerance of the monodisperse Rh_1/AC. In order to investigate the mechanism under the phenomenon, more profound research is in progress. Therefore, we deleted the content about the sulfur tolerance of the monodisperse Rh_1/AC, which maybe decentralize the highlights of the work at present.

Comments: The authors mention acetyl selectivities as low as 20%, but make no mention of the other products. The species accounting for the remaining carbon must, if possible, be enumerated and quantified. If this is not possible, then discussion of the analytical limitations is warranted. It is also essential to mention how selectivity was calculated (i.e., was it calculated based on reactant conversion or total product formation).

Response: The side product was manly methane.

Comments: The authors attribute the enhanced activity of the heterogenized Rh complex (relative to the homogeneous analog) to the AC-O ligand, with support from DFT calculations. Have the authors considered or analyzed the implications of band spreading at the Rh center upon electronic coupling to the band structure of the graphitic activated carbon surface?

Response: Introduction of strong electron donating O ligands can raise the nucleophilicity of Rh site and then accelerate the carbonylation process in homogeneous system. On the heterogenized Rh complex, O-AC species served as electron donating ligands in single Rh sites, which raise the nucleophilicity of single Rh site and then accelerate the reaction rate of oxidative addition. We indeed analyzed electronic coupling of the Rh center to the band structure of the graphitic activated carbon surface using DFT calculations. However, it is more favorable for the AC-O ligand to coordinate with the Rh center in the thermodynamics. The oxygen-containing functional

groups on the surface of activated carbon is very complicated, at present, we cannot yet expound its precise structure, here, in general, it is used O-AC to represent the oxygen-containing functional groups that cannot be discomposed during the catalyst calcination process. We are continuously trying to investigate its precise structure by means of various characterization technique, even though it is very difficult.

Comments: Please provide all specific chemicals and suppliers used in the methods section. This is essential for reproducibility.

Response: The suppliers of specific chemical used in the methods section has been supplied in the rewritten manuscript and supporting information.

Comments: Presumably, hydroiodic acid was present from the conversion of acetyl iodide to methyl acetate and acetic acid. How were the liquid products worked up prior to GC injection to remove or handle HI? This is also essential for reproducibility.

Response: Hydroiodic acid was the reaction intermediates during the reaction. However, once it was generated in the heterogeneous system, it would be consumed by methanol to form methyl iodide and water. This is a fast reaction. Therefore, the liquid product contained negligible HI prior to GC analysis. Accordingly, the heterogeneous methanol carbonylation can be actually carried out in a C-276 Hastelloy-made reactor instead of zirconium-made reactor if the methanol conversion is run at lower than 60% without corrosion for more than 3 years, while the homogeneous methanol carbonylation must be conducted in the zirconium-made reactor using the same feedstocks.

Comments: Even in ultra-high purity CO, iron pentacarbonyl is a common impurity formed upon reaction with steel cylinders during storage. Was care taken to remove $Fe(CO)_5$ from the CO stream? Carbonyl complexes are known promoters of the Cativa Ir-based catalyst, so it's conceivable that $Fe(CO)_5$ might have some effect on the Rh system. At the very least it is worth mentioning whether or not the CO stream was purified for the purposes of reproducibility.

Response: Upstream from the reactor, we have installed a purifier containing deoxidizing, decarbonylation, dehydration agents. This would guarantee that impurities in the feed gas such as $Fe(CO)_5$ were removed before entering into the reactor.

Comments: We recommend that the authors either remove discussion of industrial viability, or present a fell techno-economic analysis, comparing their process to the Monsanto and Cativa processes to support their claims about applicability.

Response: A techno-economic analysis, comparing the Monsanto and Cativa processes is a complicated work and its relevance with the purport is small. The discussion of industrial viability has been deleted.

Comments: In the EXAFS data, the Rh-Rh and Rh-I features appear essentially identical in R-space. Is it possible to reliably deconvolute these features in the fitting process, especially during the transient studies?

Response: The bond length of Rh-Rh is very close to that of Rh-I, which makes it difficult to distinguish the two bonds in R space. However, wavelet transform (WT) analysis can provide

more accurate and full-scaled information for separating backscattering atoms in both radial distance and k-space resolution. From the WT contour plots of Rh/AC and Rh_1/AC in Supplementary Fig. 4 in rewritten manuscript, it can be clearly seen that although the R values of the intensity maximum are quite similar, the k values of 8.1$Å^{-1}$ for Rh/AC and 10.9 $Å^{-1}$ for Rh_1/AC can be assigned to Rh-Rh and Rh-I coordination shell, respectively. No signal of Rh-Rh backscattering in *k* space can be observed in Rh_1/AC, which further confirms the complete transformation of Rh NPs into Rh single-atom sites.

Minor comments:

The sentence structure and grammar often distract from the science trying to be conveyed. As such, the manuscript (including its title) would substantially benefit from thorough grammatical and syntactical revisions to improve clarity.

Response: We have rewritten the manuscript and try out best to make the text more fluency and accurate.

Reviewer #2

This manuscript presents a study of a single metal site heterogeneous Rh catalyst for methanol carbonylation that form in situ upon exposure of Rh nanoparticles to a CH_3I/CO environment. Much experimental and theoretical effort was spent in identifying the structure of the single Rh atom complexes, which are shown to be much more active than their nanoparticle and homogeneous counterparts. While the results here are interesting, I do not feel that they warrant publication in Nature Communications for several reasons. First, the authors present the idea of using heterogeneous mononuclear complexes as substitutes for nanoparticle catalysts as a relatively new idea. However, researchers such as Bruce Gates have been studying such complexes for decades. As such, I do not feel that the authors have done a thorough review of the literature related to supported sinngle metal complexes. Second, this method of single atom complex stabilization with halohydrocarbons is not likely to be extendable to other catalytic reactions as methanol carbonylation actively requires and uses I ligands around the metal complex center, making this particular reaction the most ideal or perfect candidate reaction, whereas such I ligands can have negative consequences on other reactions. Third, I have several major critiques of the theoretical methods and design of the models used here (see below for details), which is used here to rationalize the structure of the catalyst and the improved performance of the single atom Rh complexes. As such, I do not recommend this manuscript for publication in Nature Communications.

Response: The referee made some constructive comments on our work. These comments are very useful to improving the manuscript. Firstly, we rewrote the manuscript. After a thorough review of the literature related to supported single metal complexes, the introduction part was thoroughly new from the points of the preparation of heterogeneous mononuclear complex catalyst and catalyst regeneration, our preparation method for heterogeneous mononuclear complexes is via

in-situ formation upon exposure of Rh nanoparticles to a mixture of CH_3I/CO, and the oxygen-containing functional groups is indispensable for the formation of mononuclear complexes, which is absolutely different from the preparation of heterogeneous mononuclear complexes by Prof Bruce Gates. Secondly, because methanol carbonylation requires I ligands around the metal complex center, making it as one of the most ideal candidate reactions for the single atom complex with halohydrocarbons. However, it does not mean that these catalysts were absolutely invalid for other reactions. For instance, a single atom nickel iodide electrocatalyst with atomically dispersed iodine ligands with excellent performance for the hydrogen evolution reaction was reported recently [*Angew. Chem. Int. Ed, DOI: 10.1002/anie.201905554*]. Furthermore, based on the latest advances of our work, the atomic dispersion of supported noble metal nanoparticles was not an individual phenomenon but a shared route to disintegrate noble metal NPs to single-metal-sites, such as Ru, Rh, Pd, Ag, Ir and Pt. Although such I ligands maybe have negative consequences on other reactions in some cases. From the point of catalyst regeneration, the desired dispersion of NPs can also be further obtained by controlling the post processing conditions of the supported mononuclear complexes and the I ligands can also be removed by proper reduction treatment. Thirdly, without the full evidences of experimental data, the sole theoretical calculations to support the reaction mechanism may be unconvincing, especially for the reaction mechanism for heterogeneous methanol carbonylation. As we all known, there are great difficulties to directly in-situ capture the dominant surface species on the activated carbon supported catalyst by infrared spectroscopy due to the fact that the transmitted or reflected infrared spectroscopy signals of the activated carbon are very poor in the in-situ cell. Therefore, in the revised manuscript, we deleted the discussion of heterogeneous carbonyaltion. Based on the comments of reviewers and the latest advances of our work, we focused on the spontaneous transformation of metal nanoparticles into the corresponding mononuclear complexes and the universality of the method for most noble metals. We will further investigate the reaction mechanism of heterogeneous methanol carbonylation according to the comments of referees and search evidential experimental data. We will detailed explain the critiques in the following response.

Theory Critiques

Comments: Besides a brief comparison in turnover frequencies and some elementary barriers, no significant comparison is made between the homogeneous Rh catalyst standard for methanol carbonylation and the HSMSCs here. No arguments are presented here why this heterogeneous system outperforms the homogeneous catalyst. What structural or charge effects from the surface immobilization lead to decreased elementary barriers and increased turnover frequencies? The inclusion of theoretical models allows for deeper understanding of the active site's structural and electronic relation to performance, and the lack of such analysis weakens this manuscript. The choice of Rh HSMSC structure for modeling the methanol carbonylation reaction pathway is questionable. First, the authors find the Rh HSMSCs exists as +3 and +4 complexes and propose structures $[Rh(CO)_2I_3(O\text{-}AC)$ and $Rh(CO)I_4(O\text{-}AC)]$, with both charge states having 6-fold

coordination. However, in Figure 5 the active complex is modeled to be $Rh(CO)_2I_2$(O-AC) with Rh having a charge of +2, with the +4 complex forming only after CH_3I oxidative addition. Under the characterization conditions, the Rh complex was exposed to the CH_3I/CO environment required for reaction to occur, so on average the characterizations should capture the dominant surface species and the theory in Figure 5 suggests this to be $Rh(CH_3CO)(CO)I_3$(O-AC), observable with in situ IR spectroscopy, where Rh is said to be +4. How does the theory justify such models in relation to the detailed experimental characterizations performed on the system? Furthermore, the proposed mechanism shows that the Rh requires transitions between +2, a rare Rh complex state, and +4. What is the role and function of the +3 Rh complex in this system? Homogeneously, the Rh complex cycles between the +1 and +3 charge states. What is the justification for the models with +2/+4 transitions for this system?

Response: In-situ EPR spectroscopy confirmed the electron paramagnetism of fresh Rh_1/AC, suggesting the presence of the Rh^{II} or Rh^{IV} species. Moreover, the intensity of paramagnetic resonance in spent Rh_1/AC catalyst was obviously stronger than that of fresh Rh_1/AC catalyst, suggesting the content increase of Rh^{IV} or the appearance of Rh^{II} after carbonylation reaction. In the classic reaction mechanism, $[Rh(CO)_2I_2]^-$ was proposed as the initial active species. The cycle between Rh^{I} and Rh^{III} species completed the reaction path. Because the rate-limiting step is oxidative addition of methyl iodide to anion species $[Rh(CO)_2I_2]^-$, as we known, the introduction of strong electron donating O or P ligands may raise the nucleophilicity of Rh site and then accelerate the carbonylation process. The coordination of O-AC species served as electron donating ligands in single Rh sites, which raise the nucleophilicity of single Rh site and then accelerate the reaction rate of oxidative addition.

In the case of the high activity of Rh_1/AC catalyst, the choice of AC support with much micropore is also very important apart from its high specific surface area and uniformly distributed single sites. The capillary forces effect of its micropore caused by relative adsorption potential superposition makes support possess a quite strong capture capability for small adsorbate molecules at low relative pressure, which exerts a unique effect on the feature of supported metals to suppress hydrogen adsorption, promote the multiple adsorption of CO and inhibit the dissociation of adsorbed CO. As an excellent adsorbing material, AC possesses an iodine value as high as 1000 mg/g, exhibit a superior adsorption capacity for iodine species. Therefore, the unique sorption character of AC significantly enhances the local concentration of CO and CH_3I on the surface around single active sites.

However, there are a few difficulties to directly in-situ capture the dominant surface species on the AC supported catalyst by IR characterization due to the fact that the transmitted or reflected IR signals of the AC support are very poor in the in-situ cell. In-situ EPR/ESR certified the paramagnetic resonance of spent Rh_1/AC catalyst, suggesting the presence of Rh^{II} or Rh^{IV} species during the carbonylation process. Due to the instability of Rh^{II} species, the ex-situ XPS analysis only detected the existence of Rh^{IV} species on the spent Rh_1/AC catalyst. Analogical to the

reaction mechanism of homogeneous counterpart, oxidative addition of methyl iodide to initial active species inevitably increased two valence state of Rh ion. Therefore, we speculated Rh^{II} species as the initial active species on Rh_1/AC catalyst. Because the heterogeneous methanol was operated under the syngas atmosphere, Rh^{II} species can be obtained via the in-situ reduction of $Rh(CO)_2I_3$(O-AC) and $Rh(CO)I_4$(O-AC) on the fresh Rh_1/AC catalyst. The apparent activation energies on Rh_1/AC catalyst and homogeneous $[Rh(CO)_2I_2]^-$ catalyst were measured. The apparent activation energy (E_a) of the Rh_1/AC catalyst is 21.1 kcal/mol, obviously lower than 35.9 kcal/mol of the homogeneous $[Rh(CO)_2I_2]^-$ catalyst. This indicates the Rh_1/AC catalyst really is intrinsically more active than the homogeneous catalyst. However, more convincing experimental data still were needed to support the mechanism proposal.

In the revised manuscript, we focused the spontaneous transformation of metal nanoparticles into mononuclear complexes and the universality of the method for most noble metals. The activated carbon supported nanoparticles of Ru, Rh, Pd, Ag, Ir and Pt metals with loading up to 5 wt. % were completely dispersed by reacting with CH_3I and CO mixture. The dispersive process of Rh nanoparticle was in depth investigated as an example. As stated above, the discussion about heterogeneous methanol carbonylation was deleted in the revised manuscript.

Comments: As this work is concerned with the in situ formation of catalytically active sites, the theoretical results here should be presented as free energies under reaction/treatment conditions. For example, under reaction conditions, what is the dominant surface complex from those presented in Figure 3? The adsorption of CO and I will see significant entropy effects due to loss of translational and rotational degrees of freedom that could significantly affect the stability of each complex and such factors are currently unaccounted for. A similar question can be asked of the effect of entropic losses on the barriers shown in Figure 5.

Response: The suggestion for the theoretical calculation was very pertinent. If we want to illustrate spontaneity of the atomic dispersion of NPs, the Gibbs free energy including the entropy effects was needed. In the current state, we can only conclude that the atomic dispersion of AC supported Rh NPs is favorable in thermodynamics when both CO and CH_3I participate concurrently in the reaction due to the strong exothermicity. To calculate the Gibbs free energy using DFT calculation now is a time-consuming process, the discussion about spontaneous dispersion in DFT calculation was changed into “Accordingly, the atomic dispersion of AC supported Rh NPs is favorable in thermodynamics when both CO and CH3I participate concurrently in the reaction due to the strong exothermicity” in the revised manuscript.

Comments: The argument against possible dimer Rh structures is made primarily from the weak interaction found to occur between two fully coordinated Rh complexes (Figure S7). However, this is measured entirely by the Rh-Rh distance in the optimized structures. What is the interaction energy between these complexes? Why were no dimer Rh complexes with I ligands bridging the metal centers tested? More work is needed in regard to fully defining the single atom nature of the complexes.

Response: Dimer Rh complexes with I ligands bridging the metal centers was possible present during the process. If this dimer Rh complexes was immobilized by the oxygen-containing group, the construction and space of two neighbouring anchoring site cannot accommodate the dimer Rh complexes. Further, we want to emphasize that a single Rh atom was detached from the parent after attacked by CO and I• free radical. There was still no Rh-Rh metal bond even in the dimer Rh complexes with I ligands bridging the metal centers, maybe have some such dimer Rh complexes with I ligands bridging the metal centers.

Comments: The formation of Rh single atom complexes from Rh nanoparticles shown in Figure S16 suggests that the O-AC site is a critical component required to form the Rh complexes. Is this true? I agree that the O-AC are likely sites for the stabilization of such isolated species, but are they required to directly participate in the transition state for breaking off at Rh atom? Furthermore, we are shown that the formation of such Rh complexes is highly favorable in Figure S16, but this does not include entropy loss for the adsorption of CO and I, as discussed above.

Response: The key species of I• radicals and CO molecule were identified to promote the breaking of Rh-Rh bonds and the formation of mononuclear carbonyls of Rh. After migration of the Rh carbonyl species on the surface of AC, they were subsequently immobilized by the oxygen-containing functional groups (O-AC). The O-AC did not directly participate in the breaking off of Rh atom from parent.

Comments: The model of the O-AC is not very well justified. Only a single oxygen functionality is tested as a possible site for stabilizing the Rh complexes, but many different oxygen functionalities exist on activated carbon. What is the rationale for this choice of functionality? What are the effects of the oxygen functionality on the stability and activity of the complex? Furthermore, the model surface itself is small, comprising only 6 ring structures. How was such a model chosen? What is the effect of surface size on the calculated energetics?

Response: Various oxygen-containing functional groups can be found on the surface of AC, such as carboxyl, lactone, anhydride, phenol, carbonyl and ether. Carboxyl, lactone and anhydride are unstable and decompose at low temperature in the form of CO_2. While the phenol, carbonyl and ether are relatively stable [*Carbon,* **37**, 1379-1389 (1999)]. Interestingly, a carbon vacancy along with either isolated phenol group or adjacent phenol-carbonyl pairs in the reduced graphene oxide surface served as anchored site for single Pd atom by atomic layer deposition [*J. Am. Chem. Soc*. **137**, 10484-10487 (2015); *Nat. Commun.* **8**, 1070(2017)]. In the AC supported single sites catalyst for heterogeneous carbonylation, a broken ether bond was proposed as the site to immobilize the single metal ion [*J. Catal.* **361**, 414-422 (2018)]. For the modeling of DFT calculation, we chose carbonyl and ether as the anchored site for mononuclear complex based on the key role of carbonyl and benzene groups on the surface of AC on the formation of mononuclear complexes. The precise structure of the O-AC is continuously investigated in our team now.

Comments: A major argument made by the authors is that these Rh complexes are good catalysts because of their resistance to H_2S poisoning, which is offhandedly argued to be due to no

opportunity for H_2S activation from fast oxidative addition. However, this argument should be improved by using the theoretical models to examine the activation of H_2S on the Rh complexes and comparing the energy pathways directly and more quantitatively.

Response: The suggestion was very useful to assess the relative tolerance of the monodisperse Rh_1/AC by using the theoretical models to examine the activation of H_2S on the Rh complexes and comparing the energy pathways directly and more quantitatively. However, in the present state, it is difficult to understand the intrinsic reason for the sulfur tolerance of the monodisperse Rh_1/AC. In order to investigate the mechanism under the phenomenon, more profound research is in progress through synthesis of some porous organic polymer of sulfur-containing functional groups to supported Rh mononuclear complexes, and test carbonylation of methanol. Therefore, we deleted the content about the sulfur tolerance of the monodisperse Rh_1/AC, which maybe decentralize the highlights of the work in previous manuscript.

Comments: Gaussian calculations used dispersion corrections, while no such corrections were included in the VASP calculations. What is the justification for not including dispersion corrections when modeling the interaction of the complex with a carbon surface fragment where such corrections are likely to be important?

Response: We agree with your idea that dispersion correlations indeed should be considered in the DFT calculation process. However, the influence of dispersion correlations is not too big. In reality, there is dispersion correlation in the model of VASP, merely the precision is not ideal as that of Gaussian calculations. In terms of dispersion correlations, the method is almost same, Gaussian utilizing the D3 modals while VASP employing D2 modal. However, as for the case of carbon materials, VASP is also a common method to optimize and calculate the geometrical structure of supported molecules.

> General Critiques

Comments: The writing in places is unpolished and casual (specifically in the introduction section). Examples can be seen in line 2 and 5 on page 3 and line 1 and 9 on page 4.

Response: Based on the comments, the manuscript was rewritten; especially the introduction part was thoroughly new from the points of the preparation of heterogeneous mononuclear complex catalyst and catalyst regeneration.

Comments: Distinct lack of references to support in the introduction. For example on page 3 second paragraph, the authors discuss previous strategies for stabilizing HSMSCs in terms of surface/support modification and claim that these previous methods do not work under “practical conditions”. However, no citations are provided to support this point. In fact, methods for modifying the surface to enable highly stable single metal sites to exist has been shown to be a viable method [Ref. 11 in the text; Science 2017, 358, 1419-1423; ACS Catal., 2019, 9, 1595-1604]. As a more minor example, on page 4 second paragraph no references are provided in relation to the Cativa and Monsanto processes.

Response: The introduction part was thoroughly new and proper references were cited to support key claims.

Comments: Figure 4a scheme is very confusing as it appears to be some type of reaction pathway, but is not a true reaction pathway as the attack by CO and I are presented in panels b-e as parallel possible reactions.

Response: Given the confusion of Figure 4a, we deleted it the revised manuscript.

Comments: Figure 5c should include high quality images of the model structures directly, instead of Lewis structure cartoons of the reactive structures.

Response: The discussion of heterogeneous methanol carbonylation was all deleted.

Reviewers' comments:

Reviewer #2 (Remarks to the Author):

The authors addressed many of the original concerns with this work by refocusing the manuscript on describing and characterizing their method for dispersing metal nanoparticles supported on activated carbon. Their thorough efforts with many different techniques (i.e. XRD, EXAFS, TEM, TPD, IR, etc.) paint a clear picture of the nanoparticle dissolution process and requirements for Rh, which is the only metal deeply characterized. Additionally, they briefly demonstrate the potential application of this method to other metals, such as Pd, Pt, Ir, Ru, and Ag. The authors present a reasonable justification for removing the originally included discussions on methanol carbonylation activity and mechanism over their single atom Rh1/AC catalyst in their response letter, and the expansion of the single atom catalyst creation method to other metals provides a useful proof of concept to readers. I recommend this manuscript for publication in Nature Communication with minor revisions (see below).

- The DFT methods section does not appear to have been updated during the review process. The calculation method of transition states is discussed, but I did not see any transition states within the revised manuscript. Additionally, if the VASP calculations did indeed use dispersion corrections as claimed in the response letter (model D2), then this should be explicitly discussed and appropriate citations included.

Reviewer #3 (Remarks to the Author):

This is without doubt an interesting paper. Clearly it has been refereed by two others who have concentrated on the reaction for which the metal is being redispersed or the theory aspect. I will concentrate on the redispersion.

I feel a central feature is the authors do not give credit for earlier work even though they reference most of the key papers. They tend to bury them as general references. The case in point is the use of references 14-16. These concern the redistribution of deactivated gold catalysts using CH3I where the process redisperses gold to give atomic species stabilized by iodine. What the current authors fail to point out that the gold catalysts are used for methanol carbonylation. Indeed there is a commercial process operated by Eastman using this technology and they use CH3I to reactivate the used catalysts and redisperse the gold (see Zoeller r, J. R.; Singleton, A. H.; Tustin, G. C.; Carver, D. L. U.S.Patents Nos. 6,506,933 and 6,509,293)
So to some extent its not surprising that this redispersion will work with other metals. In this paper the authors use CH3I and CO but surely only CH3I is needed. This needs to be a point that is answered.

My conclusion is that the authors need to give due credit to the fact that this CH3I effect has been used in industry and then the process was studied in details for the gold catalyst. Then state what is novel here which may be around the reaction being catalysed and that Rh is better than Au – but is it?

Response to reviewer #2

Comments:

The authors addressed many of the original concerns with this work by refocusing the manuscript on describing and characterizing their method for dispersing metal nanoparticles supported on activated carbon. Their thorough efforts with many different techniques (i.e. XRD, EXAFS, TEM, TPD, IR, etc.) paint a clear picture of the nanoparticle dissolution process and requirements for Rh, which is the only metal deeply characterized. Additionally, they briefly demonstrate the potential application of this method to other metals, such as Pd, Pt, Ir, Ru, and Ag. The authors present a reasonable justification for removing the originally included discussions on methanol carbonylation activity and mechanism over their single atom Rh1/AC catalyst in their response letter, and the expansion of the single atom catalyst creation method to other metals provides a useful proof of concept to readers. I recommend this manuscript for publication in Nature Communication with minor revisions (see below).

"The DFT methods section does not appear to have been updated during the review process. The calculation method of transition states is discussed, but I did not see any transition states within the revised manuscript. Additionally, if the VASP calculations did indeed use dispersion corrections as claimed in the response letter (model D2), then this should be explicitly discussed and appropriate citations included."

Response: Thanks for your valuable comments and suggestion. In the first manuscript, the transition states of Rh_1/AC catalyst for the heterogeneous methanol carbonylation

were discussed. According to the reviewers' comments, the originally included discussions on methanol carbonylation activity and mechanism over Rh_1/AC catalyst have been removed in the revised manuscript.

Currently, the contents of DFT section were updated including the newest revised manuscript and the supplementary file, and all VASP calculations were performed with DFT-D2 correction on the energies and molecular structures. Further, the related details of DFT-D2 were explicitly presented in the theoretical method section, and the citation of DFT-D2 method also is added as Ref. 50. In the Fig. S17 (a), the results indicate that the dispersion process of Rh NPs on the AC is spontaneous with the attack of CO/I• free radical. So, the reaction of dispersion about Rh NPs have no the energy barrier and corresponding transition state. As shown in the Fig. S17 (b), the potential energy curve of $Rh(CO)_2I(O\text{-}AC)$/Rh NPs complex with changed bond length as the selected bond r1/r2/r3 also presents the dispersion process without transition state. Therefore, the one-by-one dispersion mechanism based on the effective atomic number (EAN) rule of Rh NPs does not involve the transition state.

Response to reviewer #3

Comments: "This is without doubt an interesting paper. Clearly it has been refereed by two others who have concentrated on the reaction for which the metal is being redispersed or the theory aspect. I will concentrate on the redispersion.

I feel a central feature is the authors do not give credit for earlier work even though they reference most of the key papers. They tend to bury them as general references. The case in point is the use of references 14-16. These concern the redistribution of deactivated gold catalysts using CH_3I where the process redisperses gold to give atomic species stabilized by iodine. What the current authors fail to point out is that the gold catalysts are used for methanol carbonylation. Indeed there is a commercial process operated by Eastman using this technology, and they use CH_3I to reactivate the used catalysts and redisperse the gold (see Zoeller r, J. R.; Singleton, A. H.; Tustin, G. C.; Carver, D. L. U.S.Patents Nos. 6,506,933 and 6,509,293)

So to some extent it is not surprising that this redispersion will work with other metals. In this paper the authors use CH_3I and CO but surely only CH_3I is needed. This needs to be a point that is answered.

My conclusion is that the authors need to give due credit to the fact that this CH_3I effect has been used in industry and then the process was studied in details for the gold catalyst. Then state what is novel here which may be around the reaction being catalysed and that Rh is better than Au – but is it?"

Response: Firstly, we want to express our sincere thanks for your precious comments. As reported, CH_3I treatment was effective to disperse metal nanoparticles (NPs) to

smaller ones, primarily in the case of gold. Hardacre et al demonstrated that large NPs of Au supported on activated carbon (Au/AC) can be dispersed to single atom, dimer and trimer by CH_3I during the induction period of vapor methanol carbonylation reaction under 513 K and 16 bar or via bromohydrocarbons or iodohydrocarbons treatment at atmospheric pressure in the temperature range of 323 to 513 K. In addition, Hardacre et al also investigated the method of using CH_3I to disperse Au NPs supported on oxide support and discovered that there was a drop in average NPs size after the treatment (Sa, J.; Goguet, A., et al. *Angew. Chem., Int. Ed.* **2011**, 50, 8912−8916; Goguet, A. et al. *J. Am. Chem. Soc.* **2009**, 131, 6973−6975.; Sa, J.; et al. *ACS Catal.* **2012**, 2, 552−560; Morgan, K., Goguet, A. & Hardacre, C. Metal Redispersion *ACS Catal.* **2015**,5, 3430-3445). The patents of Eastman disclosed that the dispersed catalyst using CH_3I can be applied for heterogeneous methanol carbonylation (Zoeller r, J. R.; Singleton, A. H.; Tustin, G. C.; Carver, D. L. U.S.Patents Nos. 6,506,933 and 6,509,293).

Thermal treatment with methyl iodide has been proven to be a facile and successful way to redisperse NPs. However, the dispersion technique was mainly focused on Au and the improved method may be expanded to other noble metals with high loading. We try this technology to atomically disperse other noble metals and found that there was still a large portion of NPs on the Rh/AC, Ir/AC, Pd/AC, Pt/AC, Ir/AC, Ru/AC, and Ag/AC samples with 5wt% loading after N_2/CH_3I treatment. However, the treatment with CO/CH_3I mixture completely made the Rh and other noble metal NPs dispersed into the single metal atoms. We deeply sure that CH_3I is

very important to disperse NPs, but only CH_3I treatment is not enough to transform the supported NPs into atomically dispersed atom, especially at high metal loading. In the manuscript, we draw a conclusion that the combined actions of proper temperature, CO, CH_3I and oxygen-containing groups on AC were indispensable for the atomic dispersion of Rh NPs.

REVIEWERS' COMMENTS:

Reviewer #3 (Remarks to the Author):

The authors have answered the key points raised and so the paper can now be published.
